# Micelle-Assisted Lewis and Brønsted Acid Catalysis: A Review Towards Greener and Efficient Synthesis of Polycyclic and Heteroaromatic Compounds

**DOI:** 10.3390/molecules31101572

**Published:** 2026-05-08

**Authors:** Harvinder S. Sohal, Sanyojak Kanwal, Chirag G. Makvana, Navneet Kaur, Haesook Han, Manvinder Kaur, Pradip K. Bhowmik, Ankush Mehta, Kulwinder Singh

**Affiliations:** 1 Materials and Natural Product Laboratory, Department of Chemistry, Chandigarh University, Gharuan, Mohali 140413, Punjab, India; 2 Department of Chemistry, Faculty of Science, Gokul Global University, Sidhpur 384151, Gujarat, India; 3 Department of Chemistry and Biochemistry, University of Nevada Las Vegas, 4505 S. Maryland Parkway, Box 454003, Las Vegas, NV 89154, USA; 4 Chitkara School of Planning and Architecture, Chitkara University, Rajpura 140401, Punjab, India; 5 School of Engineering and Technology, K. R. Mangalam University, Gurugram 122103, Haryana, India; 6 Department of Physics, Graphic Era Deemed to be University, Dehradun 248002, Uttarakhand, India

**Keywords:** surfactant catalysis, phase transfer catalysis, organic synthesis, polycyclic aromatic compounds, green synthesis

## Abstract

Considering the expanded interest in reducing organic solvents in synthesis, surfactants and surfactant-based catalysis have been used to carry out various organic transformations in water. In recent years, the integration of Lewis and Brønsted acid catalysis with micellar systems has gained considerable attention as a powerful approach to enhance reaction efficiency while minimizing the environmental impact of synthetic processes. In this article, we depict the most recent advances in the water-interceded synthesis of different organic systems by utilizing different surfactant-type catalysts, which are important structural motifs in pharmaceuticals, agrochemicals and functional materials. Further, these methods incorporate green reaction media, mild reaction conditions, and a great yield of product with high purity in a shorter interval of time. Understanding the scope and impact of this area, authors have made efforts to collect and compile the data that indicates many named reactions, such as Friedlander annulation, aldol condensation, the Biginelli reaction, the Mannich reaction, Suzuki–Miyaura cross-coupling, etc., now take place using surfactant-based catalysts.

## 1. Introduction

In modern chemistry, one of the biggest challenges is avoiding environmental issues *like* the massive use of organic reagents in organic transformations. Out of green chemistry principles, the choice of solvent is the key problem that has driven great efforts in the development of alternative green solvents [1]. This ideal solvent should possess the environmentally beneficial properties of lessening toxicity, pollution, and energy. Out of all possible liquids, water is undoubtedly the first choice of researchers as it is selected by nature to carry out organic transformations for the synthesis of several organic composites in varied forms, *like* chemicals [2] and therapeutics [3]. Moreover, it is safe, non-poisonous, promptly accessible, inexpensive, environmentally benign, non-combustible, polar and clean compared with other hazardous solvents [4]. Furthermore, in many cases, because of hydrophobic effects, utilizing water as a solvent not only increases reaction rates but also improves reaction selectivity and reactivity, even when the reagents are partially soluble or insoluble in it [4]. Therefore, from the perspective of green science, when performing chemical reactions, water would be the ideal solvent, as it has a small impact on the environment [5].

Despite its numerous advantages, one of the major issues in utilizing water as a solvent is the low solubility of organic substances, and at times, for many substances, the catalyst is deactivated through water particles [6]. Limited dispersion of substrates often results in slower reaction kinetics and incomplete conversion, ultimately lowering catalytic efficiency. Traditionally, such limitations have been addressed by introducing organic co-solvents to improve solubility or by developing water-tolerant catalytic systems [7]. Co-solvent utilization can improve substrate solubility to some extent [8], but it compromises the environmental benefits of water-based chemistry [8,9]. Various alternative strategies, such as the development of water-tolerant Lewis acids and specially designed catalytic systems, have demonstrated their potential [10]. Nevertheless, the call for more sustainable, efficient, and universally applicable methods is still there.

Along this line, surfactants and surfactant-based catalytic systems have indeed become promising tools for mitigating the shortcomings of water as a reaction medium. Due to their amphiphilic character [11], surfactants in aqueous media become micelles, which offer hydrophobic microenvironments that are capable of enhancing catalyst stability, selectivity, and reaction rates. At the same time, Lewis acid–surfactant-combined catalysts (LASCs) and Brønsted acid–surfactant-combined catalysts (BASCs) are two types of catalysts that incorporate the catalytic function in every surfactant molecule [12,13]. Thus, surfactants have proved to be potent agents in aqueous organic synthesis because they not only raise solubility but also create catalytic microenvironments in water, which are unique.

## 2. Surfactants in Organic Synthesis

The short term for amphipathic molecules (micelle-forming molecules) is “Surface Active Agents”. The surface tension and viscosity of the solvent are changed by the addition of a surfactant in the reaction medium [10]. Due to the structural uniqueness of surfactants, they can direct almost all synthetic pathways and are nowadays extensively used in several well-known reactions [11]. Amphiphilic surfactants that will self-assemble into micelles in the water phase can improve substrate solubility and reaction rate by participating in micelle-assisted catalysis [12]. In the solution phase, surfactant molecules are self-assembled to form micelles with sizes ranging from nanometers to microns [13]. The catalytic applications of micelles were introduced in the 70s (1970s) but this concept has gained attention in the last decade under green chemistry [14]. In 2015, Lasorella [15] briefly illustrated the micellar effects on reaction kinetics and selectivity, emphasizing how micelles act as nanoreactors that enhance local reactant concentration, stabilize transition states, and provide unique microenvironments that significantly accelerate organic transformations in aqueous media.

Since micellar structure and interfacial properties strongly depend on the nature of the surfactant head group, surfactants are generally classified on the basis of the polar head group. In the case of anionic surfactants, the polar head comprises sulfate, carboxylate, sulfonate, and phosphate as the polar groups, and all these carry a negative charge. While positive charge-carrying cationic surfactants contain a nitrogen atom, the common cationic surfactants are pH-sensitive amine and quaternary ammonium-based surfactants [16]. Both positive- and negative-charge-bearing zwitterionic (amphoteric) surfactants are represented mainly by acyl ethylene diamines and alkyl amino acids. In surfactant chemistry, one of the exciting developments was introduced in the late 1980s and early 1990s by Menger [17] in the form of Gemini surfactants, which comprise two hydrophobic tails that are joined together via a spacer (mostly methylene or oxyethylene) of varied length. Uncharged nonionic surfactants comprise polyhydroxyl and polyether units as polar groups. Since the nonionic surfactant hydrophilic group is uncharged, it derives its solubility from polar polyoxyethylene or polyol groups. Lipshutz and his coworkers reported the use of nonionic surfactants, such as PTS [18], 5TPGS-750-M [19], or Nok [20], as efficient catalysts in the development of a variety of water-mediated transformations. To extend the role of surfactants beyond solubilization, acidic catalytic sites have been deliberately incorporated into amphiphilic architectures, enabling surfactants to function as both self-assembling media and catalysts. Based on the nature of the acidic functionality, these systems are broadly classified into Lewis acid–surfactant catalysts (LASCs) and Brønsted acid–surfactant catalysts (BASCs) [21], where catalysis occurs at the micellar interface. Such dual-functional catalysts facilitate acid-catalyzed organic transformations in aqueous media while minimizing or eliminating the need for organic co-solvents.

### 2.1. LASC-Based Synthesis of Organic Compounds

Since the revelation of water-tolerant Lewis acids, reactions catalyzed via Lewis acids have become feasible in water media [22]. To carry out various organic transformations, Lewis acids, *like* Yb(OTf)_3_ [23] and Sc(OTf)_3_ [24], and a few various metal salts [25] are used. As already discussed above, similarly, in the case of Lewis acid catalysis, there is also a need to apply some natural co-solvents [THF, toluene, acetonitrile] to quicken the organic reaction [26,27]. Thus, to overcome this issue, anionic surfactants are employed with a Lewis acid to form LASCs. The structure of an LASC impetus comprises a metal Lewis acid, and, to date, Sr [28], Sc [29], Cu [30], Zr [31], Al [32], Yb [33], metals, Lewis acids, surfactants, and joined catalysts have been prepared. Additionally, the Mukaiyama aldol reaction of silylenol ethers with aldehydes in water is catalyzed via Sc(OTf)_3_, with the addition of a minimum quantity of surfactant SDS in it [34]. Firstly, Kobayashi reported distinct attributes of LASCs in multifunctional catalytic properties [35], as one of the most broadly researched catalytic systems, which has attracted growing attention in many previous years. The reaction catalyzed by this new type of catalyst is prompted in water without the utilization of organic co-solvents [36]. This new kind of catalyst plays a dual role: as proposed by Kobayashi, it behaves both as a Lewis acid and as a surfactant. As a Lewis acid, it initiates the reactant molecule, and as a surfactant, it forms emulsions in water. Consequently, utilization of an LASC in the reaction shows high productivity and is an environmentally benign method. LASCs, for example, Fe(DS)_2_, Sc(DS)_3_, and Zr(DS)_4_, catalyzed a few organic reactions, including aldol, Diels–Alder, lack of hydration, allylation, Mannich-type, multi-part, and cationic polymerization.

[Sc(DS)_3_] Scandium tris (dodecyl sulfate) has been utilized by numerous analysts for catalyzing various organic changes, as it catalyzed the Michael reaction [37], Friedlander annulation for the formation of polycyclic quinolines [38], and the formation of different α-amino phosphonates [39], indoles and electron-deficient olefin conjugate addition (Friedel Crafts type) [29] in water at room temperature. [Zr(DS)_4_] catalyzed the formation of quinoxaline-imitative [31] and quinazoline-imitative [40] C-C and C-N bond-forming through Michael addition of aromatic amines or indoles to electron-deficient olefins [41] utilizing H_2_O as green media at room temperature. While “Lewis acid–surfactant-combined catalyst (LASC)” [Al(DS)_3_·3H_2_O]-catalyzed synthesis of thiiranes from epoxides and of β-amino alcohol [32] in water at room temperature has also been reported.

Scandium tris-dodecyl sulfate (STDS) [36], copper bis(dodecyl sulfate) [30], and a polymer-upheld scandium-based Lewis acid [42] have been utilized to quicken carbon coupling reactions, such as Mukaiyama aldol condensation and allylation reactions in water. However, different [LASCs] dodecane sulfonate salts [25] and Brønsted acids [43] have been utilized to increase the speed of aldol and allylation reactions in water. The allylation reaction can also be accomplished in water by the utilization of a metal–surfactant-consolidated impetus, *like* a lanthanide (III)/transition metal (II) impetus [Yb (DOS)_3_, Cu (DOS)_2_] [33]. The Mukaiyama aldol reaction in water was also catalyzed utilizing proficient surfactant amphiphilic calix [6] arene derivatives [44] and iron (II) dodecyl sulfate [45].

Several analysts have utilized different LASCs in water. For example, the “Lewis acid–surfactant-combined catalyst (LASC)” C_12_H_25_SO_3_Na/CuSO_4_ was used to prepare a 1,2-dihydro-isoquinoline imitative [46]; Cu(DS)_2_ was used to catalyze the thioacetalization and trans-thioacetalization of carbonyl compounds and O, O-acetals [47]; [Fe(DS)_3_] was used to catalyze the preparation of a chromeno[4,3-b] chromene imitative [48]; and dodecyl-sulfated silica support, as another upheld surfactant, was used to immobilize nano-TiO_2_ to form an NTDSS impetus [49]. This impetus is utilized to catalyze the combination of coumarin imidate. Ce[LS]_3_ has been utilized in the preparation of dihydropyrimidinones or thiones [50] and cerium trisdodecylsulfate for the creation of alkyl esters by dissolvable-free transesterification and esterification reactions [51].

**Scheme 1:**
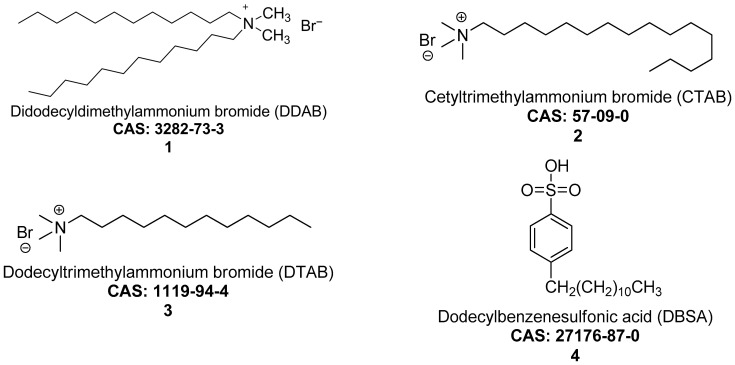

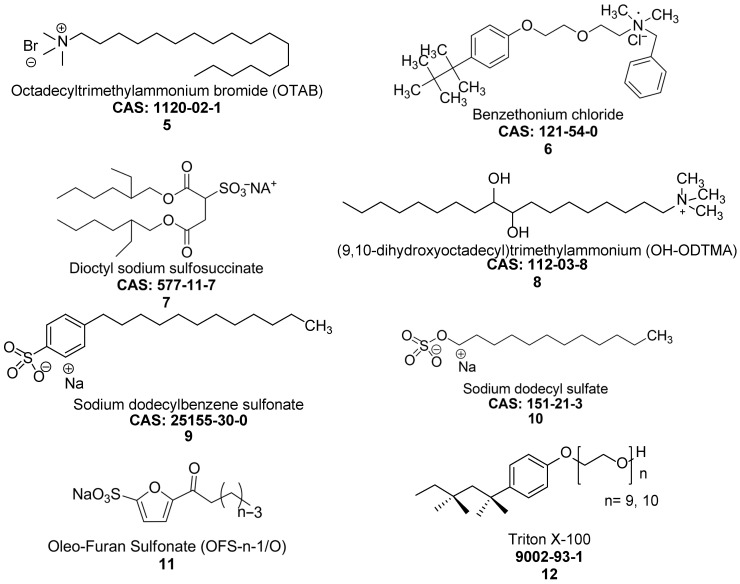
Structures of LASCs used in organic transformations.

So, “Lewis acid–surfactant-combined catalysts (LASCs)” are among the influential heterogeneous catalysts in synthetic chemistry. The research using LASCs has been summarized from time to time by researchers around the world. In 2003, Schramm and his coworkers [52] discussed the application of surfactants. In 2015, De [53] and his team published a report on natural surfactant classification and their properties and La Sorella [15] discussed advancements in organic synthesis catalyzed via amphiphilic micellar media. All the structures are shown in Scheme 1 (**1**–**13**).

#### 2.1.1. Classification of Lewis Acid–Surfactant-Combined Catalysts (LASCs)

Lewis acid–surfactant-combined catalysts (LASCs) represent a distinct category of micelle-assisted catalytic systems in which a Lewis acidic metal center is integrated with an amphiphilic anionic surfactant. This dual design allows catalysis to be done effectively in water by using both substrate activation and micellar solubilization. LASCs can be classified based on: (i) the nature of the metal center, (ii) the type of surfactant used, and (iii) their catalytic role in green aqueous transformations.

##### Classification Based on Metal Center (Lewis Acid Component)

**Rare Earth Metal-Based LASCs**

One of the most thoroughly studied classes of micellar catalysts is LASCs containing lanthanide rare earth metals, such as scandium (Sc) [29], Ytterbium (Yb) [54], Yttrium (Y), Lanthanum (La), and other lanthanides. They possess good water tolerance and high Lewis acidity, which is very beneficial for aqueous organic transformations. Enhanced catalytic performance can be explained as the combined actions between efficient carbonyl activation by the Lewis acid center and an enrichment in substrate concentration at the hydrophobic micellar core.

**Transition Metal-Based LASCs**

Since then, many transition metal-based LASCs, such as Fe(II), Fe(III), In(III) and Cu(II) salts, along with alkyl sulphate/sulphonate surfactants, have become indispensable due to their low costs and broad applications. Iron-based LASCs *like* Fe(DS)_3_ have been widely used in the synthesis of heterocycles, the synthesis of benzothiazole and the preparation of bis(indolyl)methanes [55]. Copper systems are best suited for C-C and C-N bond formation reactions, i.e., Michael addition, and the functionalization of indole. They give a better combination of economic factors, environmental effects and catalytic efficiency.

**Main Group Metal-Based LASCs**

Main group metal-based LASCs provide a lower toxicity system, are more sustainable and have moderate Lewis acidity compared to the main group metal system [56]. This method uses Zinc (Zn) and Tin (Sn) salts as sources for these main group metal-based LASCs. Zinc and Tin surfactant–aqueous conditions support the development of greener synthetic methodologies.

##### Classification Based on Surfactant Type (Anionic Component)

**Sulphate-based LASCs**

Alkyl sulphate surfactants, especially dodecyl sulphate (DS), are the most often used anionic component for LASC systems [38]. It is known that metal dodecyl sulphate complexes such as Sc(DS)_3_ and Fe(DS)_3_ are effective in the formation of micelles, in which organic substrates are encapsulated into hydrophobic cores [55]. The sulphate head group remains firmly coordinated with the metal center, which provides solubility in water. These systems showed high catalytic activity in aqueous media without any organic co-solvent.

**Sulphonate-based LASCs**

The amphiphilic and catalytic properties of LASCs with similar characteristics have also been investigated with dodecane sulphonate and other sulphonate-containing surfactants. Sulphonate surfactants usually showed high hydrolytic stability, tunability of hydrophilic–lipophilic balance [57] and high catalyst activity sustained through structural stability in aqueous media.

##### Classification Based on Catalytic Role and Application

**Hydrophobic/Water-stable Catalysts**

In aqueous media, LASCs self-assemble into micelles that generate hydrophobic domains. This leads to solubilization and portioning of organic substrates into such confined regions. Thus, this will lead to effective molarity and an enhanced reaction rate, and this structure will also prevent water from interacting with the Lewis acidic center, thus avoiding poisoning [58].

**Green Catalysts**

LASCs are seen as environmentally safe catalysts because the reaction can be carried out in an aqueous medium, thereby eliminating the use of any potentially harmful organic solvents. These systems also make product separation easier and reduce the toxicity of chemicals involved in the reaction, and the catalyst can also be reused for the next reaction without much loss of its activity; hence, these systems can be termed green catalysts [59].

### 2.2. BASC-Based Synthesis of an Organic Compound

“Brønsted acid–surfactant-combined (BASC) catalysts” are very advantageous for catalyzing organic reactions in water media. As expressed before, to reduce the use of harmful metal impurities in water media, an enormous number of researchers are involved in searching for justifiable as well as effective alternatives. Association with a [BASC] catalytic agent, for example, *p*-octylbenzenesulfonic acid (OBSA), perfluorooctanesulfonic acid, lauric acid, dodecylsulfonic acid (DSA), *p*-dodecylbenzenesulfonic acid (DBSA), and so forth, in organic transformation sometimes satisfies this goal.

**Scheme 2:**
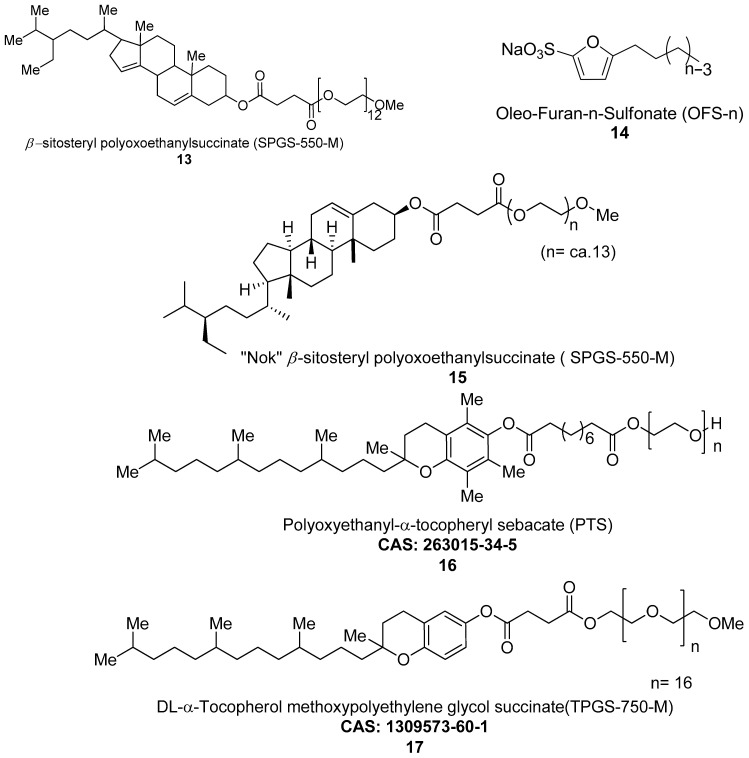

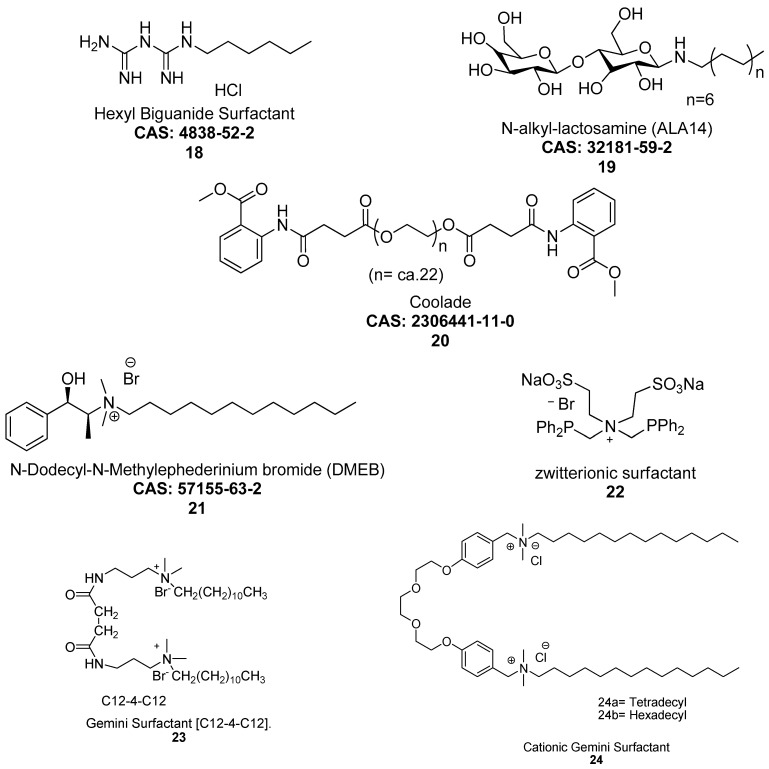
Structures of BASCs used in organic transformations.

BASCs, such as dodecyl benzenesulfonic acid, have been used to catalyze the synthesis of *α*-amino ketones [60] and a *bis*(indol-3-yl)alkane imitative in water [61]. However, *p*-dodecylbenzene sulfonic acid (DBSA) has been used to catalyze the synthesis of dihydropyrimidinone derivatives by a one-pot Biginelli reaction [62]. Further, DBSA was utilized for the preparation of 1, 3, 5-triaryl benzenes [63], and allylic alkylation of alkyl alcohol was done via [BASCs] calix[n] arene sulfonic acids in water [64]. Others revealed that the Lewis and Brønsted–surfactant-consolidated HPA impetus Cr[(DS)H_2_PW_12_O_40_]_3_ [65] is used for the productive change of cellulose into sugar, followed by the dehydration of sugar into HMF. BASCs *like* (PEG-OSO_3_H) sulfuric acid-modified polyethene glycol are used for Strecker synthesis of α-aminonitriles [66]. BASCs functionalized with [DOPA][Tos] 3-(N, N-dimethyloctylammonium) propane sulfonic acid toluene sulfate were utilized for the amalgamation of a series of toluene sulfonic acid [67]. In 2009, Shiri and coworkers reported data on different types of Brønsted acid–surfactant-combined catalysts [68]. Kaur and teammates [69], in 2018, specifically discussed *p*-DBSA as a “Brønsted acid–surfactant-combined catalyst (BASC) ”. From the literature reports, it is evident that not many efforts have been made to compile data on [BASC]-based reactions. All the structures are shown in Scheme 2 (**13**–**24**).

#### 2.2.1. Classification of Brønsted Acid–Surfactant-Combined Catalysts (BASCs)

Brønsted acid–surfactant-combined catalysts (BASCs) are amphiphilic systems in which a proton-donating acidic functionality is integrated with a surfactant structure. These catalysts simultaneously provide Brønsted acidity and micellar confinement, facilitating acid-catalyzed reactions in aqueous media. Based on the literature surveyed, BASCs can be categorized into anionic sulfonic acid surfactants, ionic liquid-based BASCs, di-cationic ionic liquid systems, and natural/bio-based BASCs.

##### Anionic Surfactant-Based BASCs

Anionic sulfonic acid surfactants such as dodecyl benzenesulfonic acid (DBSA) represent the most widely reported BASC systems. These catalysts have a strongly acidic sulphonic acid group on a long hydrophobic alkyl chain. They form micellar aggregates in aqueous media; thus, they act effectively in proton transfer and concentrate hydrophobic substrates [70]. They were successfully used in aldol concentration, Bignelli reactions, multicomponent synthesis and heterocyclic formations. As a result, they produced high yields under mild and eco-friendly conditions.

##### Ionic Liquid-Based BASCs

Ionic liquid-based BASCs are quaternary ammonium or imidazolium salt-derived systems designed with tailored hydrophobic tails and acidic counterions. An example includes 3-(N, N-dimethyloctylammonium)propane sulfonic acid toluene sulphate ([DOPA][Tos]). These catalysts exhibit the combined nature of ionic liquids (low volatility, thermal stability) and surfactants (good emulsification, interfacial catalysis), which is advantageous for aqueous organic reactions. The tunability of the structure gives them the ability to adjust acidity and micelle formation properties and accordingly control catalysis performance.

##### Di-Cationic Ionic Liquid BASCs (BASDILs)

Di-cationic ionic liquid systems, termed BASDILs, are a sub-category of BASCs that comprise acidic groups (usually sulphonic acid) and two cations, which are linked by a spacer. The example of 1,2-*bis*[N-methyl-N-(3-sulphopropyl)-alkylammonium]ethane betaines shows good thermal stability, low CMC, and recyclability [71], which may favor multiple catalysis cycles in aqueous organic synthesis.

##### Natural and Bio-Based BASCs

To support sustainable chemistry, bio-based BASCs have been developed by combining renewable surfactants such as saponins with Brønsted acids (e.g., *p*-toluene sulphonic acid). These systems are biodegradable, less toxic, and environmentally benign while retaining efficient catalytic activity. Bio-based BASCs demonstrate the potential of integrating naturally occurring amphiphiles with acid catalysis for greener organic synthesis [72].

## 3. Review of the Literature

Synthetic organic reactions for cyclic compounds have been of significant interest, particularly in water, as it is a safe, inexpensive, and clean solvent. To overcome the issue related to a traditional catalytic system for the preparation of organic compounds [heterocycles, non-heterocycles] or to avoid the use of co-solvents, environment-benign surfactants and surfactant-combined LASCs and BASCs have been developed [36]. The catalytic activity of surfactants and surfactant-combined catalysts is generally enhanced in aqueous systems compared to organic solvents. Additionally, LASCs and BASCs perform double functions; for example, as an acid, both initiate substrate particles, and as a surfactant, both form emulsions in water. Among the numerous advantages of LASCs and BASCs, the main features are magnificent yields, ecological benefits, and ease of operation, and the catalysts can be reused without any significant loss in their catalytic properties. The latest literature of the last five years on the most recent advancements in surfactant-, LASC- and BASC-catalyzed organic changes in watery media has been surveyed below.

**Scheme 3:**
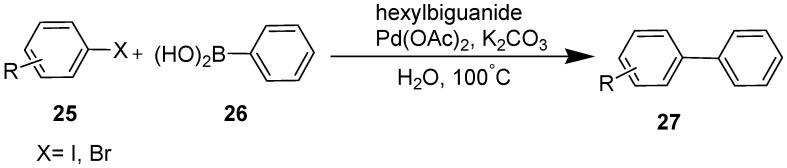
Suzuki–Miyaura reaction using a hexyl biguanide surfactant.

In 2018, Fortun et al. [73] worked on the synthesis of alkyl-biguanide/biguanidium surfactants. This newly reported Pd/hexyl biguanide surfactant is used to catalyze the reaction of aryl halide **25** and phenyl boronic acid **26**, i.e., the Suzuki–Miyaura reaction, in water at 100 °C (Scheme 3). On substitution of various aryl groups or the electron-withdrawing group in the R position using this efficient catalytic procedure, products **27** are acquired in good yield.

**Scheme 4:**
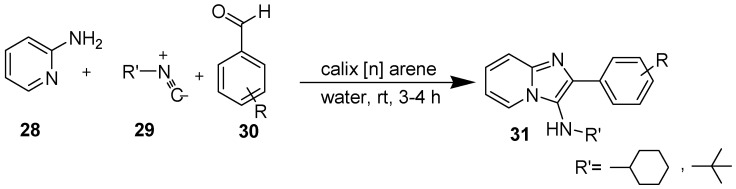
Synthesis of the imidazo[1,2-a] pyridines using calix[n]arenes.

**Scheme 5:**
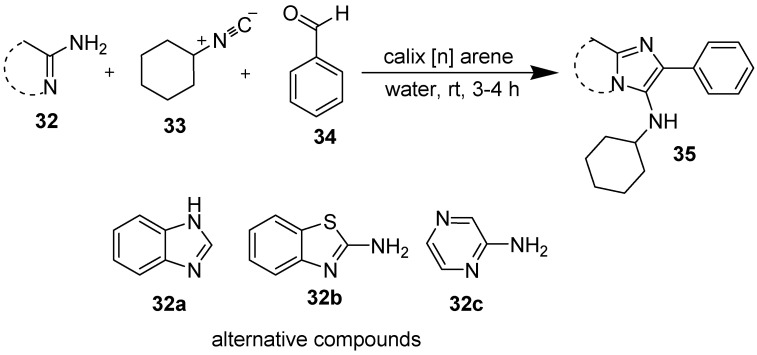
Preparation of some fused imidazole rings using Calix[6]arene-SO_3_H.

In 2018, Rostami et al. [74] revealed the synthesis of compounds **31/35** using surfactant imidazo[1,2-a] pyridines calix[n]arenes-SO_3_H. A moderate to excellent yield of product **31** is obtained via reacting isocyanides **29**, aldehyde **30**, and 2-aminopyridine **28** in the presence of a surfactant catalyst (Scheme 4). Moreover, a different range of yield from 75 to 96% in a shorter period is acquired on the substitution of different electron-withdrawing or releasing groups in the R position. However, it is also used to catalyze the reaction of fused imidazole **32**, aldehyde **34**, and isocyanides **33** to give product **35** (Scheme 5). The key highlight of this catalyst is that it can be easily recycled or reused without any noticeable change in its activity.

**Scheme 6:**
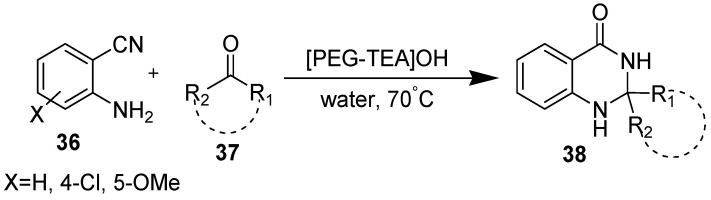
[PEG-TEA] OH-based synthesis of 2-substituted-2,3-dihydroquinazolin-4(1H)-ones.

In 2018, Safaei et al. [75] reported the use of [PEG-TEA] OH polyethene glycol-bonded tetraethyl ammonium hydroxide as a catalyst in the preparation of substituted-2,3-dihydroquinazolin-4(*1H*)-ones **38**. This catalyst is used to catalyze the reaction of carbonyl compounds **37** with 2-aminobenzonitrile **36** in water to give product **38** (Scheme 6). In this efficient procedure, a wide range of carbonyl compounds, which include aliphatic and aromatic ketones **37** and aldehydes, react with 2-aminobenzonitrile **36** and give product **38** in good to excellent yield from 70 to 95%.

**Scheme 7:**
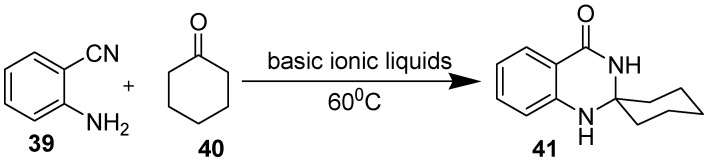
([PRIm][OH])-initiated condensation of cyclohexanone.

In 2018, Zhang et al. [76] synthesized IL-([PRIm][OH]). This 1-Propyl-3-alkylimidazole hydroxide ionic liquid is used in catalyzing the condensation reaction of 2-aminobenzonitrile **39** with cyclohexanone **40** at 60 °C (Scheme 7). A high yield of product **41** was acquired using this procedure within a short interval of time.

**Scheme 8:**
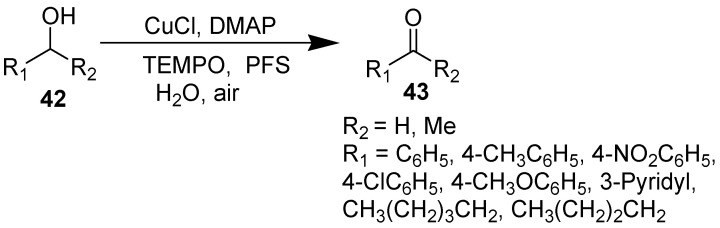
Perfluoro surfactant-based aerobic oxidation of alcohols.

In 2018, Shen et al. [77] investigated the use of a perfluoro surfactant as a cocatalyst in the aerobic oxidation of alcohols. This cocatalyst, along with catalyst CuCl/DMAP/TEMPO, is used to catalyze aerobic oxidation of alcohols **42** to ketones **43** in water (Scheme 8).

**Scheme 9:**
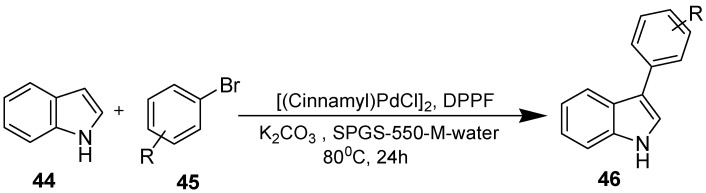
C3-arylation of indole and aryl bromide.

**Scheme 10:**
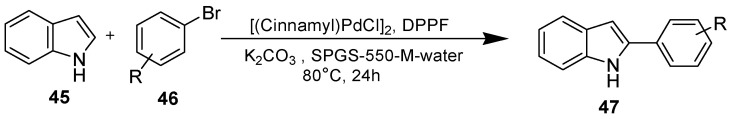
C2-arylation of aryl bromide and indole.

In 2019, Vaidya et al. [78] reported C-H arylation of indoles in water carried out using SPGS-550-M surfactant along with [(Cinnamyl)PdCl]_2_ and DPPF. Using this designer surfactant, C-3/C-2-arylation of aryl bromide **45** and indole **44** is done in the easiest way to give products **46** and **47**. However, the catalytic micellar aqueous solution can be reobtained or reused without showing any noticeable effect on product yield. The targeted products **46**, acquired via reacting indole **44** and aryl bromide **45** with different electron-releasing or electron-withdrawing groups in C-3 arylation, give a higher yield from 52 to 80% (Scheme 9). In a similar manner, in C-2 arylation, products **47**, obtained by reacting indole **44** with aryl bromide **45** carrying different electron-releasing/withdrawing groups, also give a good yield, i.e., from 71 to 76% (Scheme 10).

**Scheme 11:**
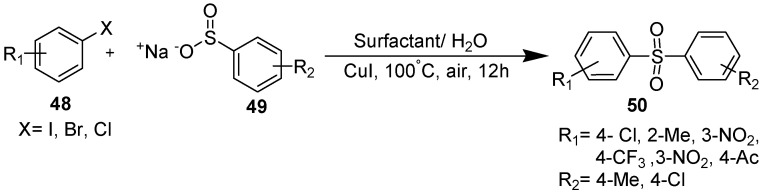
Ullmann C-N couplings in ALA14 aqueous solution.

In 2019, Ge et al. [79] revealed the preparation of a sugar-based surfactant, alkyl lactosamine ALA14 **19**. This surfactant **19** plays a dual function by providing a micellar environment for the aggregation of substrates and by acting as a ligand. The structure of this naturally degradable catalyst comprises a lactose-based hydrophilic part and a long alkyl chain-based hydrophobic part. The C-X coupling reaction of compounds **48** and **49**, carried out using this efficient catalyst **19** in water, gives products **50** in good to excellent yield (Scheme 11). Further, the catalyst, along with the catalytic micellar aqueous system, can be easily recycled.

**Scheme 12:**
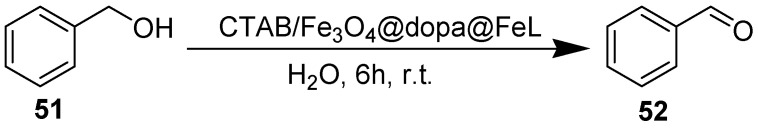
Nanocatalyst-initiated oxidation of alcohol.

In 2019, Chakraborty et al. [80] worked on the synthesis of nanocatalyst CTAB/Fe_3_O_4_@dopa@ML and also showed its use in catalyzing the oxidation of alcohols **51** to keto **52** groups at room temperature in an aqueous medium (Scheme 12). This nanocatalyst can be easily recycled five times without any noticeable change in its effectiveness.

**Scheme 13:**
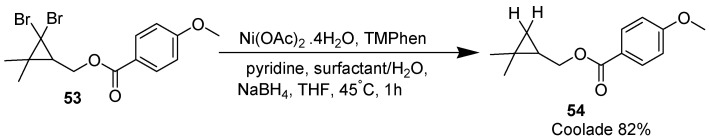
Coolade-catalyzed double reduction of **54**.

In 2019, Lee et al. [81] reported an advantageous, easily accessible, low-foaming “Coolade” surfactant **20**. Catalyst **20** is used to catalyze several gas-evolving reactions; for example, it is used in the double reduction of gem-dibromocyclopropane **53** to **54** in the presence of water (Scheme 13). This advantageous surfactant plays a major role in solving the issue of this reduction, i.e., the production of a massive amount of foam due to the evolution of hydrogen gas. Using this catalyst in the double reduction of gem-dibromocyclopropane **53** solves this issue as it possesses low-foaming properties while carrying out the reaction in micellar aqueous conditions.

**Scheme 14:**
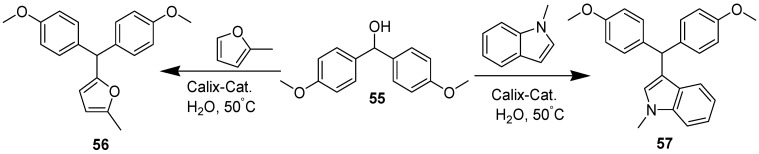
Amphiphilic calix[n]arene-initiated coupling reaction.

**Scheme 15:**
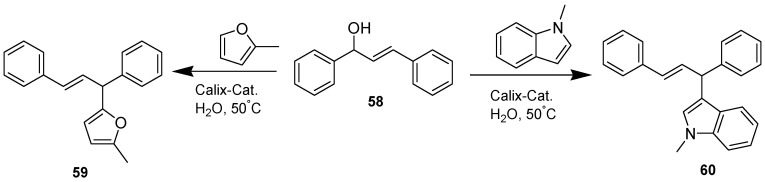
Amphiphilic calix[n]arene-initiated coupling reaction.

In 2016, Sayin et al. [82] synthesized amphiphilic calix[n]arene derivatives and also showed their use as a catalyst in a coupling reaction (Scheme 14). This efficient catalyst is used in the coupling reaction of two activated sec-alcohols **55** and **58** with 2-methylfuranto give products **56** and **59**, while reaction with N-methylindole gives **57** and **60** (Scheme 15).

**Scheme 16:**
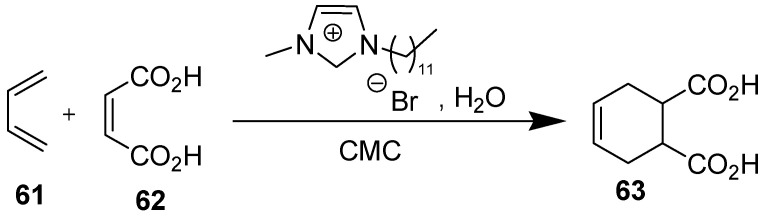
IL-initiated Diels–Alder reaction.

In 2019, Soffietti et al. [83] reported the synthesis of ionic liquids (ILs) based on 1-alkyl-3-methylimidazolium cations. These ionic liquids act as a surfactant, and also, they are used to catalyze the Diels–Alder reaction of diene **61** and dienophile **62** in water conditions (Scheme 16). The yield of cycloadduct **63** obtained by this effective method is good. So, this catalytic micellar system that is used for cycloaddition between **61** and **62** is beneficial for cycloalkene **63** development.

**Scheme 17:**
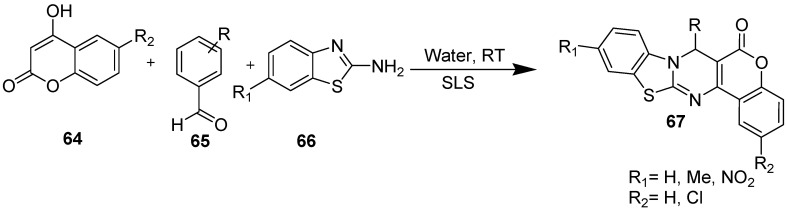
Synthesis of chromeno[4,3-d]benzothiazolo[3,2-a]pyrimidin-6(7H)-one.

In 2016, Sahu et al. [84] investigated the preparation of fused pyrimidines **67** via using sodium lauryl sulphate as a catalyst in a reaction of 4-hydroxycoumarin **64**, benzaldehyde **65**, and 2-amino benzothiazole **66** in an aqueous medium (Scheme 17). Moreover, using this environmentally friendly method, the resultant products **67** are acquired in good to excellent yields of 81–95%.

**Scheme 18:**
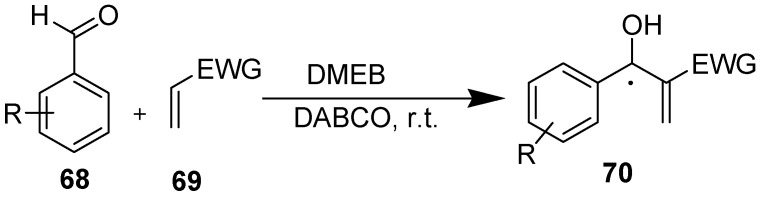
(DMEB)-catalyzed MBH reaction.

In 2016, Shairgojray et al. [85] revealed that (DMEB) **21** a cationic chiral surfactant can be used to induce asymmetry in the Morita–Baylis–Hillman reaction. This catalyst, with further aid of DABCO, catalyzed the reaction of acrylonitrile **69** and aromatic aldehyde **68** in water to give the resultant product **70** (Scheme 18). The products targeted using this methodology are acquired in good to moderate yield, i.e., from 68 to 78%.

**Scheme 19:**
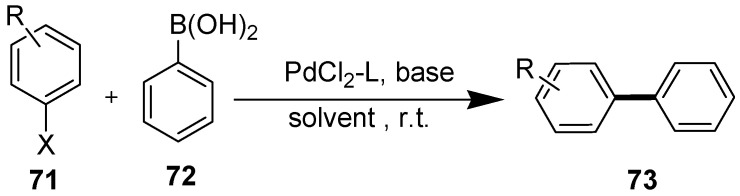
SM cross-couplings.

**Scheme 20:**
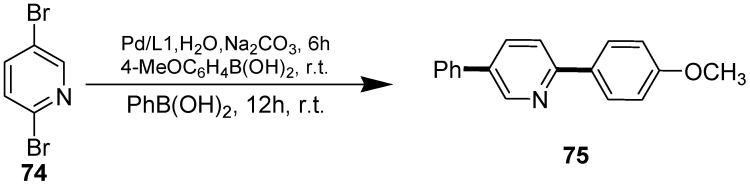
Diarylation of 2, 5-dibromopyridine.

In 2016, Qiu et al. [86] reported the SM (Suzuki–Miyaura) cross-coupling reaction using a Pd catalyst with a bidentate phosphine-type zwitterionic surfactant in water at room temperature. The reaction of aryl halides **71** (R = EDG or EWG) with arylboronic acids **72** formed several biaryls **73** in moderate to excellent yields of 78–96%, with similar efficiency for heteroaryl substrates (79–93%) (Scheme 19). The system also enabled regioselective diarylation of 2,5-dibromopyridine (Scheme 20), affording high yields with selectivity at the C-2 position.

**Scheme 21:**
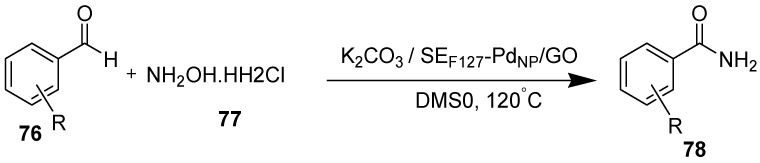
SEF127-Pd(nanoparticle)/GO-initiated synthesis of primary benzamides.

In 2016, Rostamnia et al. [87] investigated the effect of several polyethene glycol-type (PEG-type) polyethers, including PEG-300, F127, and P123, on the catalytic activity of Pd/rGO in the reaction of aldehyde **76** (R = EWG or EDG), hydroxylamine hydrochloride **77**, and potassium carbonate to give benzamide **78** (Scheme 21). The result obtained showed that among polyethers, F127 showed better results in raising the catalytic power of Pd(nanoparticle)/GO by the exfoliation of GO sheets. This catalytic system was successfully applied as a relatively green, recyclable, and efficient system in cascade primary amide **78** synthesis and single-pot amidation. Using this efficient procedure, targeted products **78** with different electron-withdrawing or electron-releasing groups are obtained in good to excellent yields of 61–92%. Some of the highlights of this catalyst are that it is highly active, efficient, and environmentally benign in one-pot conditions with recyclability at least for eight runs.

**Scheme 22:**
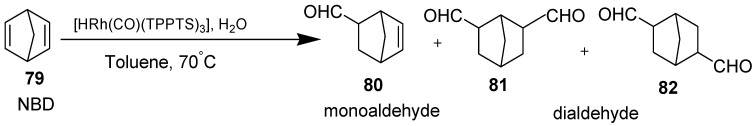
Hydroformylation of 2,5-norbornadiene (NBD).

In 2016, Yang et al. [88] reported that 2,5-norbornadiene (NBD) **79** hydroformylation using HRh(CO) (TPPTS)_3_ in an organic/aqueous biphasic system gives monoaldehyde **80** and dialdehyde **81** products (Scheme 22). The observed results showed that the cationic surfactant had a greater impact on hydroformylation in a biphasic system. Moreover, this NBD **79** hydroformylation carried out in a biphasic system under mild conditions showed high selectivity/activity to the resultant product dialdehydes **81** and **82**.

**Scheme 23:**
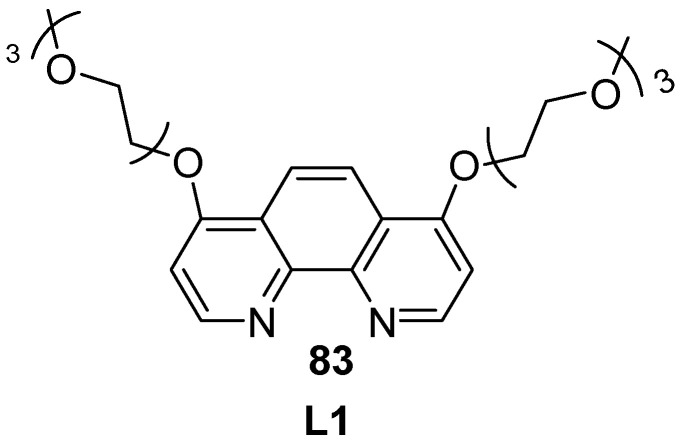
PEG-functionalized amphiphilic nitrogen ligand L1.

**Scheme 24:**
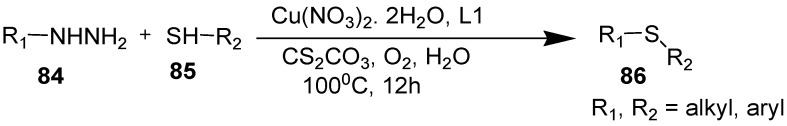
Surfactant-type-catalyst-initiated aerobic oxidative cross-couplings.

In 2019, Ren et al. [89] investigated the preparation and catalytic use of an efficient surfactant-type catalyst, i.e., **83** (Scheme 23) [L1] PEG-functionalized nitrogen ligands, in aerobic oxidative coupling of thiols **85** and aryl/alkyl hydrazine **84** to compound **86** (Scheme 24). The catalytic aqueous mother liquor can be utilized up to five times without any noticeable change in its effectiveness. Moreover, the products resulting from this Cu–surfactant catalysis show excellent yield.

**Scheme 25:**
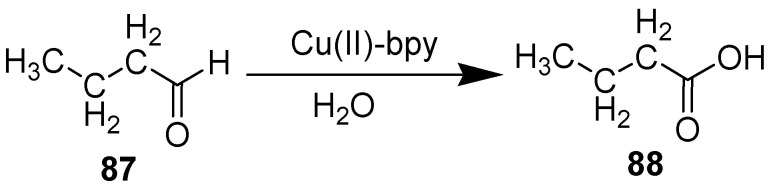
Oxidation of butanal to butyric acid.

In 2016, Mondal et al. [90] explained that a hetero-aromatic nitrogen base (bipyridine) in combination with surfactants [Cu (II)-bpy] as a catalyst can be used to enhance the rate of oxidation of butanal **87** to butyric acid **88** in water (Scheme 25).

**Scheme 26:**
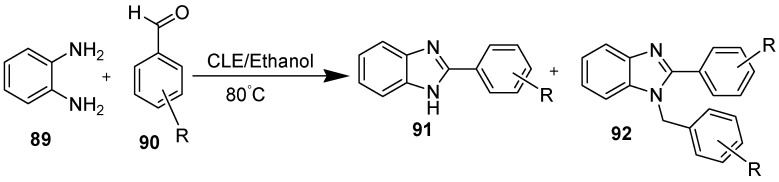
Synthesis of benzimidazoles.

**Scheme 27:**
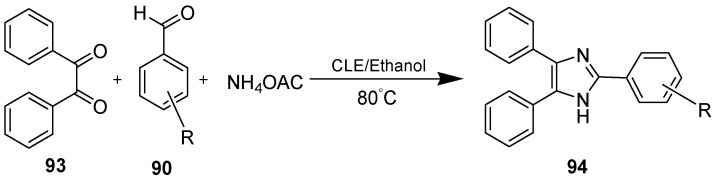
Synthesis of 2, 4, 5-trisubstituted imidazole derivatives.

In 2017, Morbale et al. [91] prepared 2-aryl-1-arylmethyl-*1H*-benzimidazoles by employing a CLE biosurfactant as a catalyst. This catalyst catalyzes the reaction of aromatic aldehydes **90** with o-phenylenediamine **89** at 80 °C (Scheme 26). The reaction of *o*-phenylenediamine **89** with various aromatic aldehydes **90** containing electron-withdrawing/releasing groups gives products **91** and **92** in good to excellent yield. Moreover, reaction feasibility is also checked on several aromatic aldehydes **90** with ammonium acetate and a 1,2-dicarbonyl compound (benzil) **93** (Scheme 27). The acquired results of this reaction procedure showed a good yield of products **94**, from 81 to 92%.

**Scheme 28:**
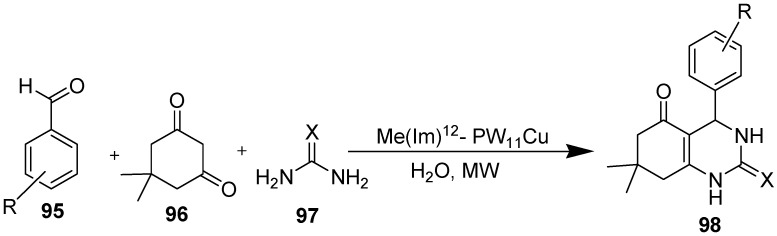
Me(Im)12-PW11Cu-catalyzed preparation of octahydroquinazolinone.

In 2016, Mozafari et al. [92] synthesized octahydroquinazolinone derivatives **98** via using a surfactant-type polyoxometalate-based organic–inorganic hybrid as a catalyst in a reaction of dimedone **96**, urea **97**, and substituted (R = H, 3-OMe, 3-Cl, 4-NO_2_, 4-Cl) benzaldehyde **95** (Scheme 28). On employing several substituents, which included different aryl/heteroaryl-containing electron-withdrawing and electron-releasing groups in the R position of aldehyde **95**, the desired product **98** can be obtained in good yield, i.e., from 87 to 90%.

**Scheme 29:**
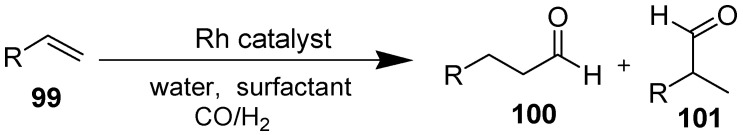
Hydroformylation of 1-dodecene.

In 2017, Pogrzeba et al. [93] reported a nonionic surfactant used to accelerate the rhodium-catalyzed hydroformylation of 1-dodecane **99** to compounds **100** and **101** (Scheme 29). The efficient catalyst structure and hydrophilicity (degree of ethoxylation) have a strong impact on the reaction rate or catalytic reaction performance.

**Scheme 30:**
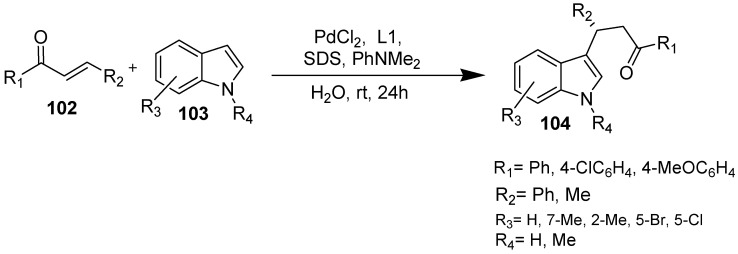
C–H functionalization of indoles.

In 2016, Kitanosono et al. [94] reported the specific nature of chiral palladium(II) catalysis with the aid of an additive or an anionic surfactant 10 in C-H indole functionalization. Indole **103** electrophilic palladation with **102** through C-H bond functionalization was accomplished in a very enantioselective way in water (Scheme 30). A long range of substituents with aryl/heteroaryl-containing electron-withdrawing or electron-releasing groups attached to indoles **103** give the resultant products **104** in good to excellent yields of 25–93%.

**Scheme 31:**
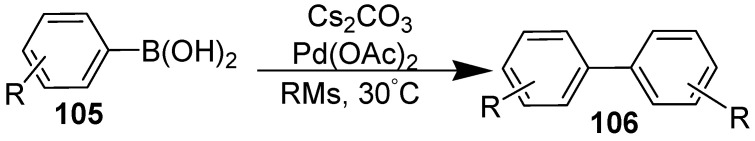
Homo-coupling of different aryl boronic acids.

**Scheme 32:**
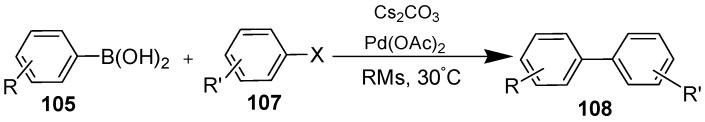
Suzuki–Miyaura cross-coupling.

In 2017, Kar et al. [95] reported that C-C homo/cross-coupling reactions proceed faster when using anionic water/AOT/Cy RM as an efficient reaction medium. The homo-coupling reaction of heteroaryl boronic acid and aryl boronic acid **105** with different electron-releasing or electron-donating groups in an aromatic gives symmetrical and unsymmetrical biaryl **106** yields from 53 to 91% (Scheme 31). Similarly, Suzuki–Miyaura cross-coupling of different aryl halides (X = Cl, Br, I) **107** with Phenylboronic acid **105** gives the desired product **108** at a yield of 62–82% (Scheme 32).

**Scheme 33:**
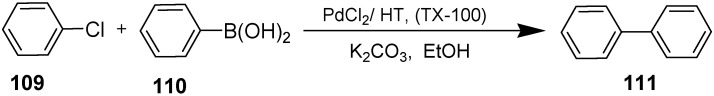
Surfactant-initiated SMC reaction.

In 2017, Choudhary et al. [96] confirmed that an efficient catalyst, a hydrotalcite-supported palladium catalyst (PdCl_2_/HT), with the aid of surfactant **12**, when used to catalyze the reaction of chlorobenzene **109** and phenyl boronic acid **110** (Suzuki–Miyaura coupling reaction), attained the desired product **111** in a high yield up to 96% (Scheme 33). In comparison with using bare (PdCl_2_/HT) as a catalyst, this yield is reduced to 69%.

**Scheme 34:**
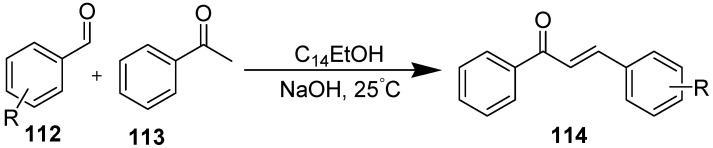
Cross-condensation (Claisen–Schmidt reaction) in micellar catalysis—NaOH/water–NaOH.

In 2019, Hafidi et al. [97] examined the catalytic efficacy of different polar groups carrying three (C1_4_EtOH, C1_4_iPrOH, C1_4_PrOH) cationic surfactants in the condensation reaction (Claisen–Schmidt) between an aromatic aldehyde (R = 4-CH_3_, 4-OCH_3_, 4-Cl) **112** and acetophenone **113** (Scheme 34). The observed result on the effectiveness of the catalytic activity in the NaOH–micellar system showed that compound C1_4_EtOH gives resultant product **114** in good yield up to 80%, followed by C1_4_iPrOH and C1_4_PrOH.

**Scheme 35:**
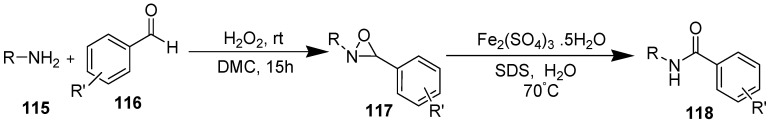
Preparation of N-alkylbenzamides.

In 2017, Kraïem et al. [98] investigated an efficient method for synthesizing N-alkylbenzamides **118** via the formation of 2-alkyl-3-aryloxaziridines **117** from benzaldehydes **116** and N-alkylamines **115**, followed by their rearrangement using Fe(III) sulfate in water with a surfactant. The *in-situ* formation of a Lewis acid–surfactant-combined catalyst enhanced the reaction rate. Oxaziridines were obtained in high yields (87–99%), while the final amides were produced in good yields (74–94), regardless of alkyl substitution (Scheme 35).

In 2018, Xu et al. [99] investigated the synthesis of Gemini surfactant [C1_2_-4-C1_2_] **23** and its role in visualizing the kinetics of hydrolysis reaction. Due to the phenomenon of high surface activity, it can easily form micelles. Better micellar catalysis efficiency for the hydrolysis reaction of 4-nitrophenyl acetate is shown by this newly reported surfactant, with lower one- or two-order values of CMC and γCMC than the old CTAB surfactant.

**Scheme 36:**
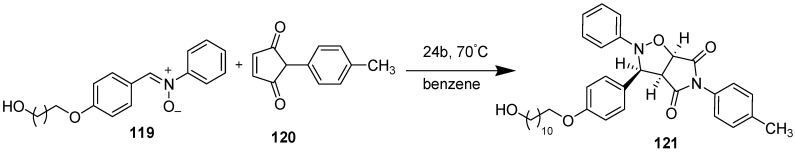
Cationic Gemini surfactant-initiated preparation of isoxazolidine.

In 2018, Öztürk et al. in [100] prepared a newly designed cationic Gemini surfactant **24b** linked to a hydrophilic oligo-oxyethylene spacer group. This surfactant **24b** had a great impact on the catalyzation of cycloaddition reactions [3+2] of compounds **119** and **120** using benzene as a solvent to give targeted isoxazolidine product **121** (Scheme 36).

**Scheme 37:**
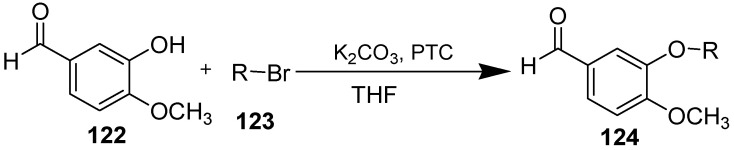
Gemini surfactant-initiated alkylation of isovanillin.

In 2016, Boz et al. [101] investigated the use of Gemini surfactants to catalyze the alkylation of isovanillin **122** with alkyl halide (cyclopentyl or octyl group) **123** in the presence of THF and K_2_CO_3_ to yield the desired product **124**. The advantages of this strategy are that it is environmentally benign and the catalyst is of low cost (Scheme 37).

**Scheme 38:**
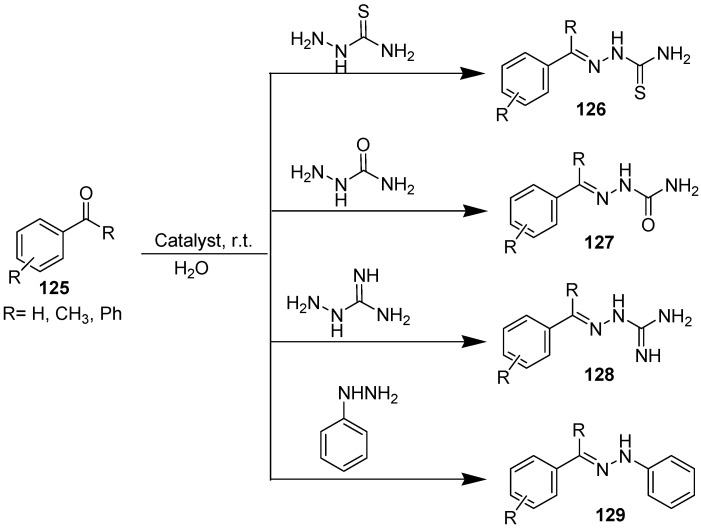
Preparation of aryl-hydrazones.

In 2016, Chavan et al. [102] reported the use of an aqueous extract of *Acacia concinna* pods as a natural-type surfactant in the preparation of aryl-hydrazones. This efficient catalyst is used to catalyze the reaction of a variety of carbonyl compounds **125** with thiosemicarbazide, semicarbazide, aminoguanidine, and phenyl hydrazine in shorter reaction times in water at room temperature to yield desired products **126**, **127**, **128** and **129**, respectively (Scheme 38).

**Scheme 39:**
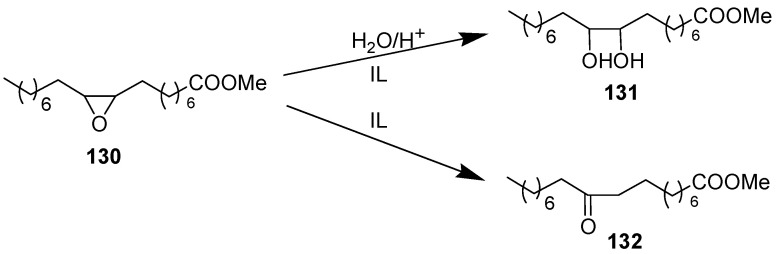
Oxirane ring-opening reaction.

In 2016, Nowicki et al. [103] synthesized amphiphilic 1-alkyl-3-methylimidazolium hydrogen sulfate ionic liquids. They act as a cocatalyst in the oxirane ring-opening reaction of **130** in epoxidized fatty acid methyl esters **131** and **132** (Scheme 39).

**Scheme 40:**
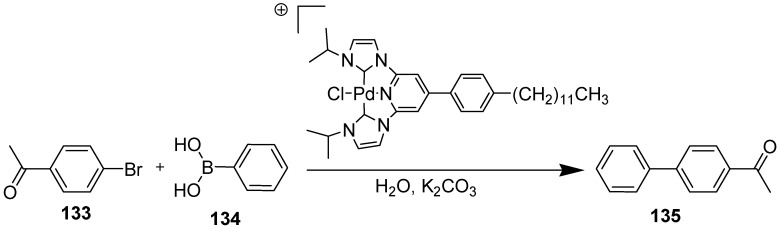
Surfactant-containing NHC moiety-catalyzed Suzuki coupling reaction.

In 2017, Donner et al. [104] worked on the synthesis of a new type of surfactant that contains an NHC moiety as a head group. Further, it also showed high catalytic activity in the Suzuki coupling of phenylboronic acid **134** with 4-bromoacetophenone **133** in water to yield the desired product **135** in great yield (Scheme 40).

**Scheme 41:**
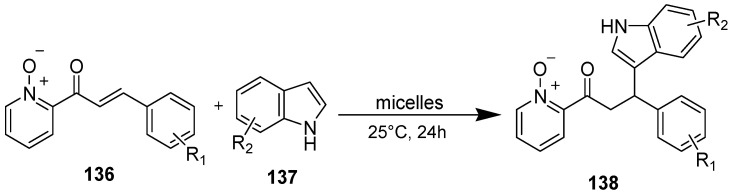
Metallo-micellar catalyst-catalyzed Michael addition reaction.

In 2020, Liang et al. [105] described a surfactant-type Metallo-micellar catalyst that was prepared and employed in the Michael addition reaction of aromatic 2-enoylpyridine-1-oxides (R_1_ = EWG, EDG, heteroaryl) **136** and indoles (R_2_ = H, 5-F, 5-Cl, 5-Br, 5-OMe, 5-COOMe, 6-Cl) **137** in water (Scheme 41). The desired products **138** are acquired in high yield, 88–96%, using this catalyst.

**Scheme 42:**
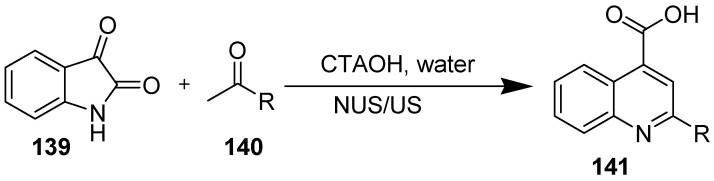
CTAOH initiated the preparation of quinoline-4-carboxylic acid derivatives.

In 2017, More et al. [106] illustrated the use of a surfactant, cetyltrimethylammonium hydroxide, in the Pfitzinger reaction. Using this catalyst, the desired product, 2-substituted quinoline-4-carboxylic acid **141**, is acquired in good yield (75–95%) via reacting isatin **139** with acetophenone **140** in water under ultrasonic irradiation (Scheme 42).

**Scheme 43:**
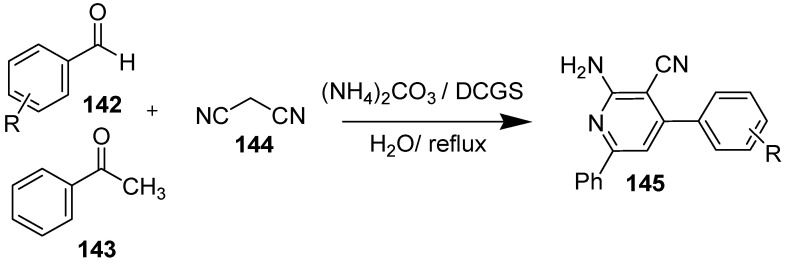
(DCGS) based synthesis of aminocyanopyridines.

In 2020, Tammadon et al. [107] worked on the synthesis of a diester cationic Gemini surfactant (DCGS) and also on its utilization as a catalyst in the synthesis of aminocyanopyridine **145**. The catalysis reaction of acetophenone **143**, malononitrile **144**, (NH_4_)_2_CO_3_, and aldehydes **142** in water to yield the targeted product in good yield, i.e., from 85 to 96% (Scheme 43).

**Scheme 44:**
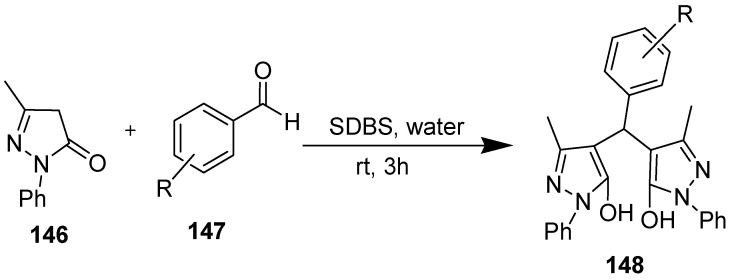
Preparation of 4, 4′-arylmethylene-bis(1-phenyl-3-methyl-5-pyrazolones).

In 2018, Zheng et al. [108] reported that an efficient catalyst, sodium dodecyl benzene sulfonate 9, can be used to catalyze the reaction of aromatic aldehydes **147** with 1-phenyl-3-methyl-5-pyrazolone **146** in water for the preparation of 4, 4′-arylmethylene-bis(1-phenyl-3-methyl-5-pyrazolones) **148** (Scheme 44). A large number of substituted aldehydes (R = EWG, EDG and heteroaryl) **147** produced **148** with a yield from 88 to 94%. The key features of this methodology are selectivity, high yield and mild reaction conditions.

**Scheme 45:**
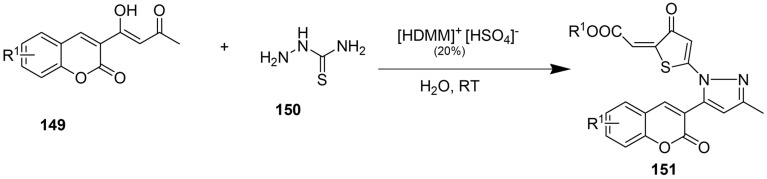
Preparation of thiazolyl-pyrazole-chromen-2-one.

In 2025, Patil et al. reported a Brønsted acidic surfactant, hexadecyl methyl morpholinium hydrogen sulphate ([HDMM]^+^[HSO_4_]^−^), as an efficient micellar microreactor for one-pot three-component synthesis of thiazolyl-pyrazole-chromen-2-one derivatives **151** in water [109]. Using a 20 mol% catalyst at room temperature, the reactions of 3-acetyl-4-hydroxy-2H-chromen-2-one derivatives **149**, thiosemicarbazide **150**, and dialkyl acetylene dicarboxylates afforded products in good to excellent yields (up to 90%) within short times, with broad substrate tolerance (Scheme 45). The enhanced activity was attributed to micelle-assisted solubilization and bond formation, and the catalyst was recyclable for up to five cycles with minimal efficiency loss.

**Scheme 46:**
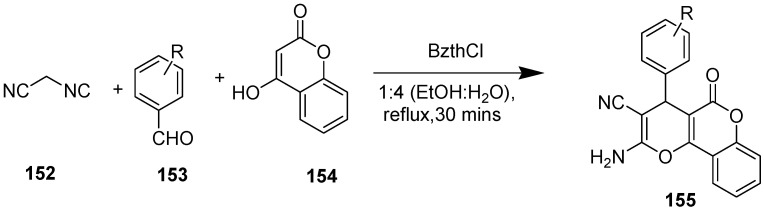
Preparation of pyrano[3,2]chromene derivatives.

In 2023, Parthiban et al. reported that benzethonium chloride (BzthCl) is an efficient bi-tailed cationic surfactant catalyst for a one-pot three-component synthesis of pyrano[3,2]chromene derivatives **155** via condensation of malononitrile **152**, substituted benzaldehydes **153**, and 4-hydroxycoumarin **154**. The reaction proceeded smoothly in a green 1:4 EtOH/H_2_O medium under reflux, affording 13 pyranochromene derivatives in good to excellent yields (77–96%) within 30–75 min [110]. Both electron-donating and electron-withdrawing substituents (R = H, halogens, NO_2_, OH, CH_3_, OCH_3_, furyl, thiophenyl) were well tolerated. The enhanced catalytic activity was attributed to micelle formation by BzthCl, which promoted the Knoevenagel–Michael–cyclization sequence efficiently under mild conditions (Scheme 46).

**Scheme 47:**
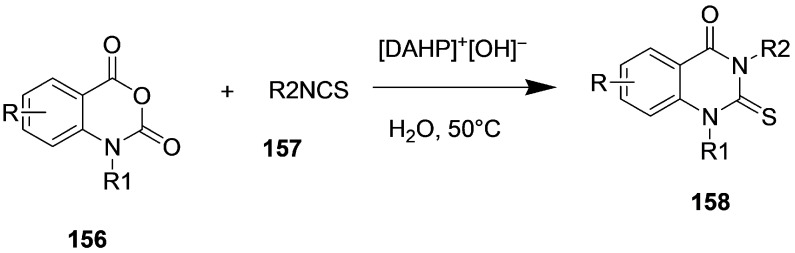
Preparation of 2-thioxo-2,3-dihydroquinazolin-4(1H)-ones.

In 2022, Patil et al. investigated the catalytic efficiency of 4-(dimethylamino)-1-hexadecylpyridinium hydroxide [DAHP]^+^[OH]^−^, a Brønsted basic surfactant, as a nano-micellar reactor for the production of 2-thioxo-2,3-dihydroquinazolin-4(1H)-ones **158** in water. The reaction between isatoic anhydrides **156** and isothiocyanates **157** was carried out in an aqueous medium at 50 °C using a 20 mol% surfactant, affording the desired products in an efficient yield (up to 81%) within short reaction times (Scheme 47). Due to effective micellar solubilization and Brønsted basic activation, [DAHP]^+^[OH]^−^ demonstrated higher catalytic activity among several common surfactants (SDS, CTAB, Triton X-100, SDOSS) [111]. The study demonstrates the micellar system’s effectiveness as a green and sustainable nanoreactor, with the catalyst maintaining its activity over four consecutive recycling cycles.

**Scheme 48:**
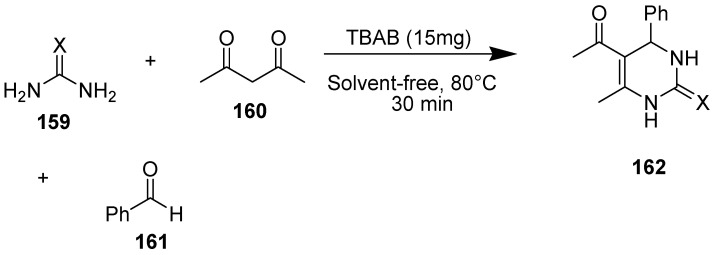
Preparation of substituted 3,4-dihydropyrimidine derivatives.

In 2024, Zolfaghari et al. investigated the efficient use of the cationic surfactant tetrabutylammonium bromide (TBAB) in the Biginelli reaction for synthesizing 3,4-dihydropyrimidine derivatives **162**. The solvent-free reaction of aromatic aldehydes **161**, β-dicarbonyl compounds **160**, and urea/thiourea **159** at 80 °C (15 mg TBAB) yielded 12 derivatives in high yields (85–97%) within 30 min. Electron-withdrawing substituents enhanced yields compared to EDGs (Scheme 48). The high efficiency was attributed to TBAB creating a homogeneous ionic medium and activating the carbonyl group, highlighting its eco-friendly catalytic role [112].

**Scheme 49:**
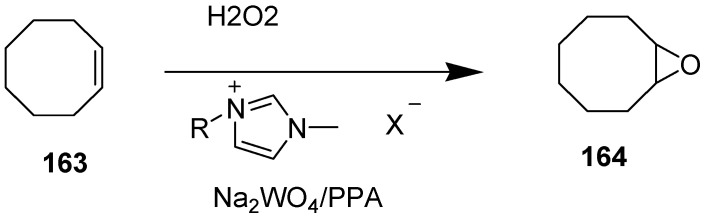
Preparation of cis-cyclooctene oxide.

In 2025, Luibl et al. studied fluorinated surface-active ionic liquids (FSAILs) as micellar phase transfer catalysts for biphasic epoxidation of cis-cyclooctene **163** using aqueous H_2_O_2_ and sodium tungstate to prepare cis-cyclooctene oxide **164** (Scheme 49). The FSAILs formed micelles that enhanced substrate solubilization and interfacial mass transfer, with catalytic activity strongly influenced by alkyl chain length and anion type. Dodecyl-substituted systems, particularly [DoMIm][BF_4_] and [DoMIm][PF_6_], provided near-complete conversions and high selectivity, while [NTf_2_] analogues were less active due to lower stability [113]. The catalysts preferentially resided in the organic phase, enabling interfacial epoxidation and facile recycling with sustained selectivity, demonstrating the efficiency of FSAIL-based systems in green epoxidation.

**Scheme 50:**
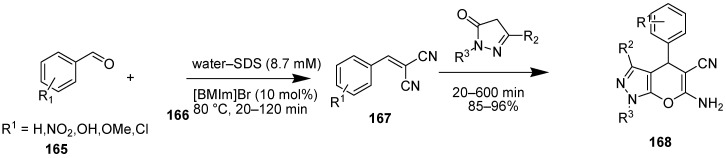
Preparation of spiro[indoline-3,4′-pyrano[2,3-c]chromene]-2-one.

In 2024, the authors reported an efficient one-pot sequential multicomponent synthesis of spiro[indoline-3,4′-pyrano[2,3-c]chromene]-2-one derivatives **168** using SDS (8.7 mM) and [BMIm]Br (10 mol%) in water at 80 °C. Substituted aromatic aldehydes **165** (R^1^ = H, NO_2_, OH, OMe, Cl), malononitrile **166**, and (5,5-dimethyl-1,3-cyclohexanedione) **167** with pyrazolone derivatives underwent Knoevenagel condensation, Michael addition, and cyclization to afford the desired products in excellent yields (85–96%) within 20–120 min (Scheme 50). The enhanced catalytic performance was attributed to the synergistic effect of SDS micelles, which improved substrate solubilization, and the ionic liquid, which facilitated intermediate activation, demonstrating an efficient and green micellar system for heterocycle synthesis [114].

**Scheme 51:**
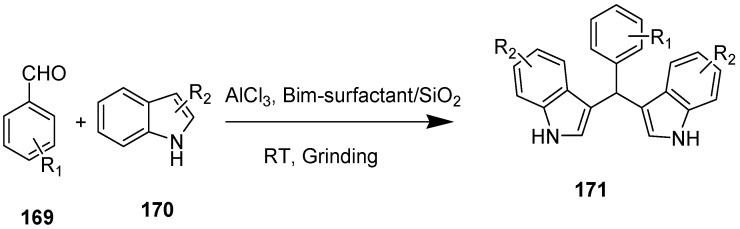
Preparation of bis(indolyl)methane.

In 2025, Khandare et al. developed a Lewis acid–benzimidazolium-based ionic liquid surfactant–SiO_2_-combined catalyst (LABimSC) for the green synthesis of bis(indolyl)methane **171** under solvent-free and dry-grinding conditions at room temperature. The LABimSC system efficiently activated aldehydes **169** toward electrophilic substitution with indoles **170**, affording 16 derivatives in 15–20 min with higher yields (83–99%) (Scheme 51). AlCl_3_.6H_2_O (0.1 mmol), surfactant (0.3 mmol) and 0.5 g of SiO_2_ support were used in the reaction [115]. The catalyst was proven to be highly recyclable and useful for gram-scale synthesis, with a good green profile, working without the use of solvents and any harmful reagents. Efficient water-assisted vesicle formation in situ and carbonyl activation by carbocation were the reasons for the high efficiency, which exhibited good performance of mechanochemical surfactant-assisted catalysis.

**Scheme 52:**
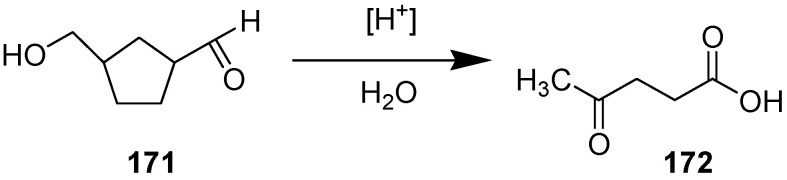
Preparation of 5-hydroxymethylfurfural (5-HMF).

In 2021, Qieo He et al. synthesized a sulfonated carbon-based solid acid catalyst (AC-SO_3_H) using a serial sulfhydrylation–sulfonation of activated carbon for the dehydration of 5-(Hydroxymethyl) cyclopentane-1-carbaldehyde **172** into 5-hydroxymethylfurfural (5-HMF) **173** (Scheme 52). This catalyst possesses high acid density, excellent thermal stability, and abundant –SO_3_H groups, which enables effective fructose transformation in DMSO. The highest 5-HMF yield (100%) and good selectivity could be achieved under optimal conditions: 65 weight% H_2_SO_4_ sulfonation, 120 °C for 2 h, and a ratio of catalyst to 5-(Hydroxymethyl) cyclopentane-1-carbaldehyde of 1:1. The AC-SO_3_H catalyst could be separated easily and showed good reusability. It could achieve about 94% yield even after four cycles. These excellent catalytic performances were ascribed to the appropriate surface acidity and porous nature of AC-SO_3_H, which illustrates that sulfonated carbon material is an attractive solid acid catalyst for biomass transformation [116].

**Scheme 53:**
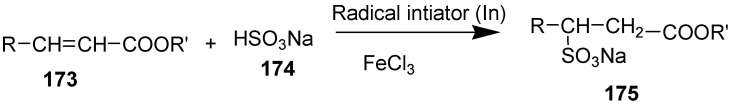
Preparation of branched-chain methyl oleate sulfonate.

In 2025, Attanatho et al. reported a surfactant-assisted sulfonation of methyl oleate **173** using sodium bisulfite **174** in a two-phase system. With TBPB as the initiator and FeCl_3_ as the cocatalyst, the addition of the branched-chain methyl oleate sulfonate MOS **175** surfactant significantly enhanced performance by reducing interfacial tension and improving phase dispersion (Scheme 53). Optimized conditions gave >80% yield after 10 h at 40 °C, whereas the reaction without surfactant was slower due to mass transfer limitations. The improved efficiency was attributed to microemulsion formation and micellar solubilization, highlighting the importance of interfacial engineering [117].

**Scheme 54:**
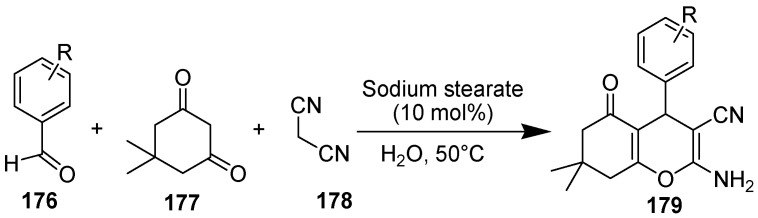
Preparation of branched-chain methyl oleate sulfonate.

In 2023, Mohamadpour et al. conducted a micellar catalytic study of sodium stearate as a combined Lewis base–surfactant catalyst for the one-pot three-component synthesis of tetrahydrobenzo[*b*]pyranes in water. Dynamic sodium stearate micelles (10 mol%) were used, and at 50 °C, the condensations of aromatic aldehydes **176** with malononitrile **178** and dimedone **177** gave corresponding tetrahydrobenzo[*b*]pyranes **179** in high yields (82–93%) in good reaction times (Scheme 54). Compared with blank and non-aqueous solvents, a low conversion rate was observed [118]. This can be attributed to the fact that the micellar structure can provide a hydrophobic reaction medium, activating substrates and inducing their cyclization in water.

**Scheme 55:**
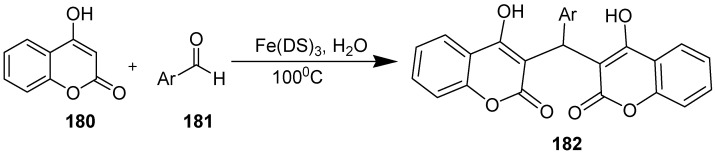
Preparation of biscoumarins and tetrakiscoumarins using Fe(DS)_3_.

In 2019, Mahmoodi et al. [119] described the preparation of biscoumarins **182** through a reaction of 4-hydroxycoumarin **180** and various aldehyde **181** catalyzed by Fe(DS)_3_ [Iron(III) dodecyl sulfate] in water at 100 °C. An extensive variety of aldehydes **181** with electron-withdrawing or electron-releasing groups in the benzene ring reacts with 4-hydroxycoumarin **180** to give a yield from 55 to 91% (Scheme 55). The substituted di-halogenated compounds exhibited great antibacterial action against Gram-positive (*M. luteus* and *S. aureus*) and Gram-negative (*P. aeruginosa* and *E. coli*) bacterial strains.

**Scheme 56:**
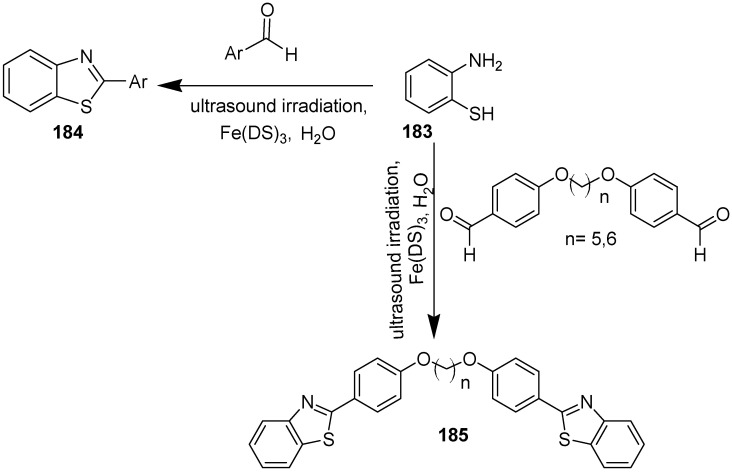
Synthesis of mono- and bis-benzothiazoles using Fe(DS)_3_.

In 2016, Pirbasti et al. [55] described the LASC Fe(DS)_3_-catalyzed synthesis of benzothiazoles by a reaction of 2-aminothiophenol **183** with different aromatic aldehydes under ultrasound irradiation in water. The benzothiazole products (**184** mono- and **185** *bis*-benzothiazoles) of this surfactant-catalyzed reaction are obtained in great yields up to 94%. The advantageous attributes of this methodology are that it is environmentally friendly and requires a short reaction time with great yields. A broad spectrum of aldehydes, heteroaryl, and bis-aldehydes, with electron-withdrawing or electron-releasing groups connected to the benzene ring, react with 2-aminothiophenol **184** to give yields from 82 to 94% (Scheme 56).

**Scheme 57:**
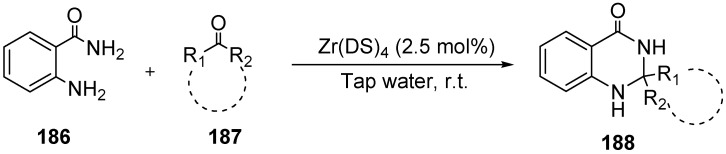
Synthesis of quinazoline derivatives.

In 2015, Safaei et al. [40] worked on the synthesis of a quinazoline derivative **188** through a [Zr(DS)_4_]-catalyzed condensation reaction of carbonyl compounds **187** and 2-aminobenzamide **186** in water at room temperature. Various ketones and aldehydes **187** react with 2-aminobenzamide **186** to give a great yield of products from 83 to 97% (Scheme 57). In comparison with the old methodology, this new surfactant-combined methodology shows significant advantageous features, such as catalyst recycling, great yields, and short reaction times.

**Scheme 58:**
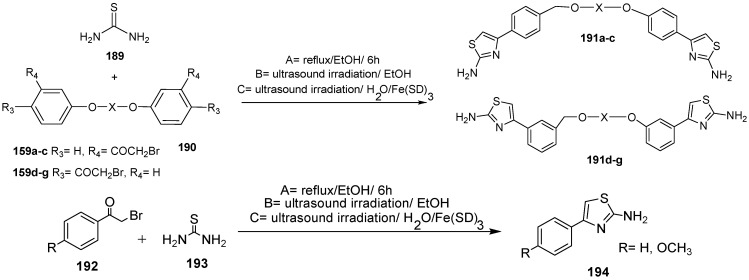
Synthesis of bis-thiazoles.

In 2017, Parvizi et al. [120] showed a novel route for the synthesis of bis-thiazoles **191**. Bis-thiazole **191** synthesis is carried out by a reaction of compound **190**, **189** thiourea, and **192** using the LASC Fe(DS)_3_ under ultrasound irradiation in water (Scheme 58). The product yield obtained by this method is brilliant, up to 87%. In correlation with the reflux strategy, this technique is exceptionally effective because of the simple setup, great yields, safety, and shorter reaction times.

**Scheme 59:**
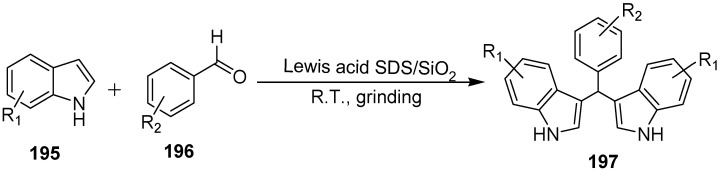
Synthesis of bis(indolyl)methane using Lewis acid–surfactant.

In 2019, Wu et al. [121] worked on the synthesis of indolyl methane derivative **195** by a reaction between indole **193** (R_1_ = H, 2-methyl, 3-methyl) and aldehyde **194** (R_2_ = NO_2_, Br, Cl, CN) catalyzed by LASCs under grinding at room temperature. The desired products **195** of this efficient procedure are obtained in great yields (Scheme 59). The key highlights of this methodology are great yields, a simple workup, and the use of a non-hazardous solvent, making this strategy more environmentally benign.

**Scheme 60:**
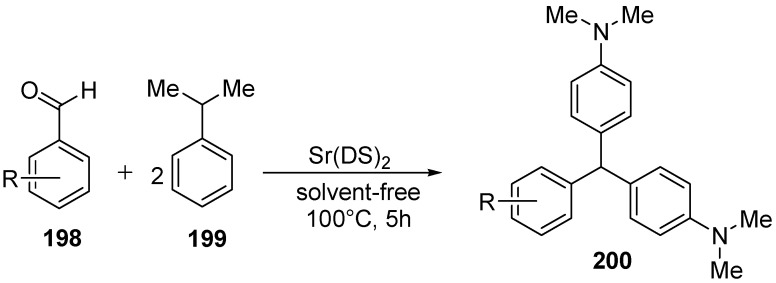
Synthesis of 4,4′-diaminotriarylmethane using Sr(DS)_2_.

In 2018, Behbahani et al. [51] developed Sr(DS)_2_-catalyzed synthesis of 4,4′-diaminotriarylmethanes **200**. The Sr(DS)_2_-catalyzed reaction of aryl aldehyde **198** with N, N-dimethyl aniline **199** was performed in an oil bath at 100 °C (Scheme 60). By substituting different electron-releasing or electron-withdrawing groups in the benzene ring of aldehyde **196**, product **198** is acquired in yields from 70 to 94%. The key highlights of this technique are simple separation and recuperation, high activity or selectivity, and great yields.

**Scheme 61:**
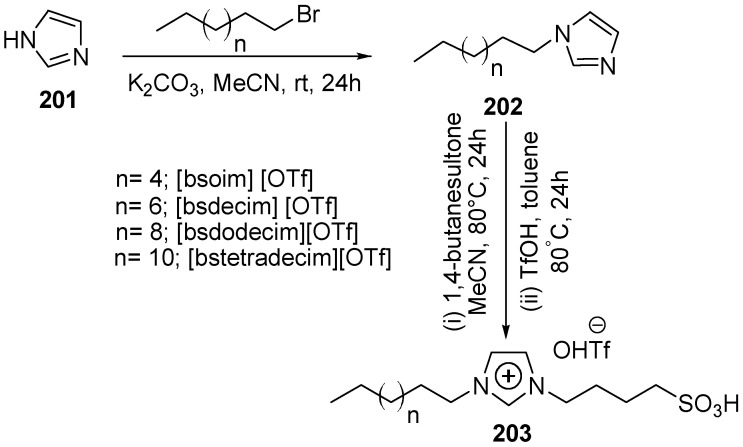
Synthesis of BAILs.

In 2018, Hulnik et al. [122] reported an emulsion cation polymerization with an LASC for the preparation of bio-based [poly-(β-myrcene, β-myrcene-co-styrene)] **203**. Higher-molecular-mass polymers [50 to 150 kg/mol] were obtained in (co)polymeric form in 10–14 h with a water-dispersible Lewis acid–surfactant-consolidated impetus (LASC) from naturally occurring β-myrcene **201** and **202** as the parent source. Here, the catalyst from the reaction mixture is easily recycled with no observable change in its effectiveness (Scheme 61).

In 2020, Destephen et al. [123] reported a new Lewis acid–surfactant hb-DBSNa/YbCl_3_ used for cationic polymerization of vinylic monomer in water. He reported that the polymerization of these monomers did not follow the cationic pathway in water; instead, these reactions proceeded via a radical pathway. Radical species identification via spin trapping experiments combined with (ESR) shows that this cationic polymerization proceeds via a radical pathway.

**Scheme 62:**
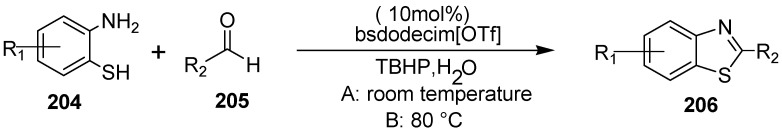
Synthesis of 2-substituted benzothiazole in water.

In 2018, Senapak et al. [124] investigated the synthesis of 2-substituted benzothiazole **206** using [bsdodecim][OTf] as a catalyst. The preparation of 2-substituted benzothiazoles **206** is carried out by a reaction of 2-aminothiophenol **204** with aldehyde **205** using catalyst [bsdodecim][OTf] in water at room temperature (Scheme 62). On substituting different alkyl, aryl, heteroaryl and electron-withdrawing or electron-releasing groups within the benzene ring in the R_2_ position of aldehyde **205**, up to 98% is obtained.

**Scheme 63:**
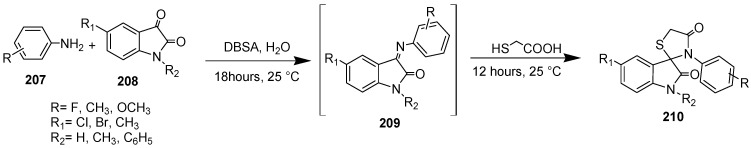
Preparation of 5-chlorospiro[indoline-3,2′-thiazolidine]-2,4′-dione.

In 2016, Preetam et al. [125] reported that spiro[indoline-3,2′-thiazolidinones] **210** are formed by the DBSA-catalyzed reaction of aniline **207** and chloroindolin-2,3-dione **208** in water at room temperature (Scheme 63). This reaction results in the formation of a Schiff base **209**, which, on further addition of thioglycolic acid, gives the desired product **210**. The product **210** yields obtained by this methodology are excellent, from 75 to 88%. When electron-withdrawing groups are attached to isatin groups, the yield obtained is higher than that when electron-releasing groups are attached to isatin. This synthetic procedure exhibits a few points of interest, for example, the use of water as a solvent, good to brilliant separated yields, and short reaction times.

**Scheme 64:**
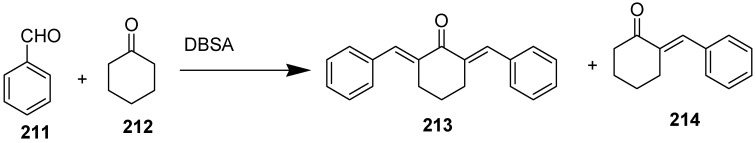
Preparation of 5-chlorospiro[indoline-3,2′-thiazolidine]-2,4′-dione.

In 2024, Abraham et al. studied the micellar catalytic efficiency of 4-dodecylbenzenesulfonic acid (DBSA) in the acid-catalyzed cross-aldol condensation of benzaldehyde **211** and cyclohexanone **212** in water to give product 2,6-Dibenzylidenecyclohexanone **213** and 2-Benzylidenecyclohexanone **214** (Scheme 64) [126]. Dynamic DBSA micelles at lower concentrations (37.5 mM) showed superior catalytic activity, giving 81% conversion with up to 96% selectivity toward the di-condensation product (ABA), whereas higher concentrations (>75 mM) resulted in reduced efficiency due to stable micelle and vesicle formation. The enhanced performance was attributed to dynamic micelles providing a large reactive interface and accessible H^+^ ions, highlighting the critical role of micellar structure in aqueous acid catalysis.

**Scheme 65:**
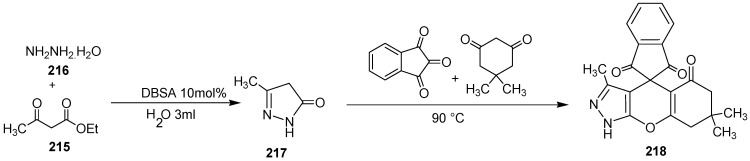
Synthesis of a tricyclic spiropyran derivative.

In 2015, Mukherjee et al. [127] developed DBSA-catalyzed synthesis of tricyclic 4-spiro pyrano[2,3-c]pyrazole **218**. This Brønsted acid–surfactant-catalyzed reaction of hydrazines **216** and ethyl acetoacetate **215** in water at room temperature forms a pyrazolone derivative **217**. Then, this pyrazolone derivative **217** reacts with 1,3-dicarbonyl and forms the desired product **218** (Scheme 65). On substitution of different aryls and the electron-withdrawing and electron-releasing groups within the benzene ring to pyrazolone **217**, yields of the desired product are obtained from 75 to 96%. Broad substrate variety, great yield, operational effortlessness, absence of hazardous reagents, easy workup, and use of an environmentally friendly catalyst are the remarkable highlights of this strategy.

**Scheme 66:**
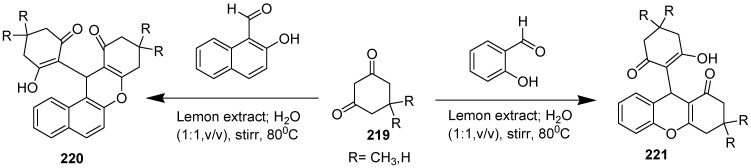
Synthesis of benzopyran.

In 2015, Morbale et al. [128] described the preparation of benzopyran **220** and **221** from the reaction of salicylaldehyde, cyclic 1,3-diketones **219**, and 2-hydroxy naphthaldehyde using a biosurfactant [lemon extract] in water at 80 °C (Scheme 66). Various electron-withdrawing or electron-releasing groups attached to carbonyl compound **219** react with 2-hydroxy naphthaldehyde and salicylaldehyde to give products yielding 96–84%. In contrast with regular strategies, this synthetic technique exhibits some significant attributes, such as the use of inexpensive, easily accessible, and environmentally benign catalysts, high yield, and the prevention of the use of hazardous reagents.

**Scheme 67:**
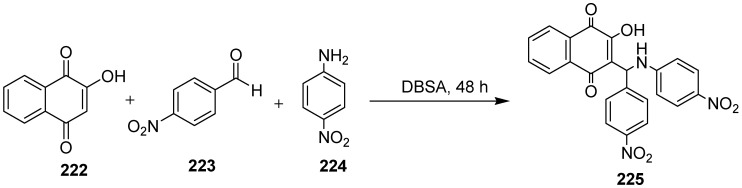
Synthesis of amino naphthoquinones.

In 2015, Filho et al. [129] worked on the synthesis of amino naphthoquinones **225** using (BASC) dodecyl benzenesulfonic acid (DBSA). DBSA catalyzed the reaction of 2-hydroxy-1,4-naphthoquinone **222**, *p*-nitrobenzaldehyde **223**, and *p*-nitroaniline **224** in water at room temperature to give the desired product **225**. Substitution of an aryl and an electron-withdrawing group on aldehyde **223** in the BASC-catalyzed reaction gives a yield of products up to 85%. While in the case of electron-releasing groups, this yield is reduced by 13%. The exhibited highlights of this approach that are appropriate for green chemistry are that it [BASC] is non-hazardous, inexpensive, and easily accessible (Scheme 67).

**Scheme 68:**
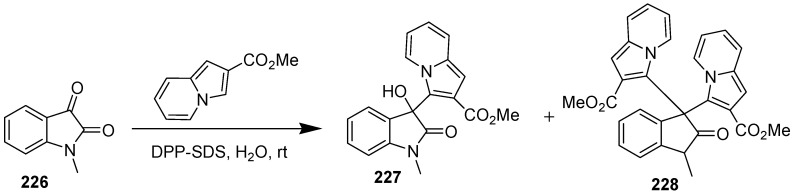
Synthesis of 1,2-addition-type of the Friedel–Crafts reaction.

In 2020, Guidotti et al. [130] reported the synthesis of 3-hydroxy-3-indolizinyl-2-oxindoles scaffolds by performing a Friedel–Craft alkylation reaction between isatin **226** and indolizine using Brønsted acid [DPP]diphenyl phosphate and SDS as a catalyst in water. Products **227** and **228** are obtained in excellent yield up to 99% by this surfactant-catalyzed methodology (Scheme 68).

**Scheme 69:**
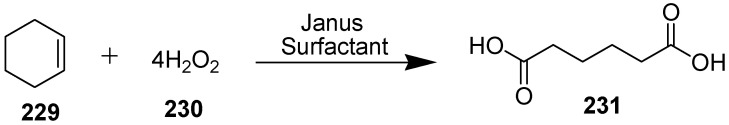
Oxidation of cyclohexene to adipic acid using a Brønsted acid surfactant.

In 2020, Vafaeezadeh et al. [131] prepared a Brønsted acid–Janus surfactant from anisotropic Janus-type material. This Brønsted acid–Janus surfactant was used to catalyze the oxidation of cyclohexene **229** with hydrogen peroxide **230** to adipic acid **231**. Other advantages of this catalyst are that it can also be used for the preparation of diethyl phthalate and a carboxylic acid imitative (Scheme 69). Furthermore, it can also be used for the oxidation of cyclohexanone and cyclopentene. This catalyst can easily be recycled via simple filtration without any noticeable change in its effectiveness.

**Scheme 70:**
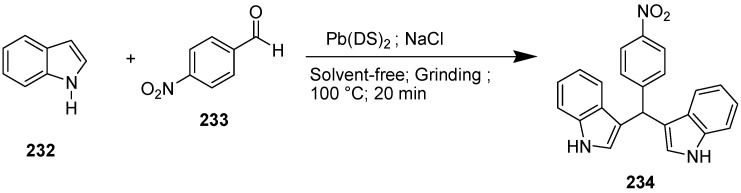
Preparation of bis(indolyl)methane.

In 2025, Wu et al. reported a mechanochemically synthesized Pb(DS)_2_ Lewis acid surfactant as an efficient catalyst for solvent-free organic transformations under grinding conditions. The catalyst promoted the condensation of indole **232** with aromatic aldehydes **233** to give bis(indolyl)methane **234** in excellent yields (up to 98%) within 20 min at 100 °C using NaCl as a grinding aid (Scheme 70). It also efficiently catalyzed cyclo-condensation of *o*-phenylenediamines with 1,2-dicarbonyls to form quinoxalines and three-component Biginelli reactions to afford dihydropyrimidinones in efficient yields (79–90%). The catalyst showed high recyclability, highlighting its potential for green mechanochemical heterocycle synthesis [132].

**Scheme 71:**
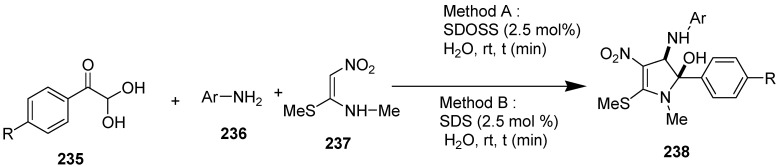
Mannich-type condensation reaction for the production of β-amino alcohol products.

In 2024, Saigal et al. examined the catalytic efficacy of anionic surfactants, sodium dodecyl sulphate (SDS) and sodium dioctyl sulfosuccinate (SDOSS), in an in-water three-component Mannich-type condensation reaction between substituted α-hydroxy ketones **235**, aromatic amines **236**, and nitro-olefin derivatives **237** to afford *β*-amino alcohol products **238** (Scheme 71). With 2.5 mol% SDS or SDOSS, the reactions were effectively conducted in water at room temperature, producing the intended products in short reaction times with efficient yields [133]. A comparison indicated that the catalytic efficiencies of SDOSS were greater than those of SDS due to the better micellar aggregation ability of SDOSS and, consequently, the enhanced solubility of hydrophobic reactants in aqueous media. This implied that the micelle-mediated catalytic effects effectively promoted multicomponent reactions under environmentally benign and mild conditions.

**Scheme 72:**
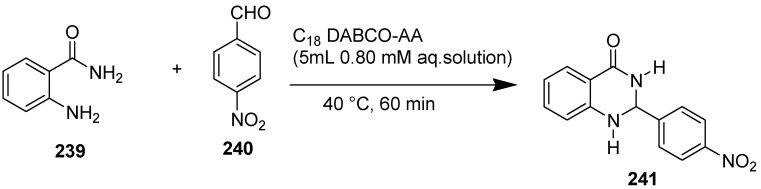
Green synthesis of 2,3-dihydroquinazolin-4(1H)-ones.

In 2024, Rajmane et al. investigated a Brønsted acidic Gemini surfactant, C18-DABCO-AA, which was used as an effective micellar catalyst to carry out the green synthesis of 2,3-dihydroquinazolin-4(*1H*)-ones **241** using anthranilamide **239** and aromatic aldehydes/ketones **240** in water (Scheme 72). Due to its low CMC (0.80 mM) and high acidity (pKa~3.3), this reaction was performed under mild conditions (40 °C), yielding products in high yield (84–96%) in a short reaction time (60–70 min). When the two sites could be reacted (using a dibasic agent), a *bis*-DHQ derivative product was obtained in 92% yield. The catalyst was recyclable up to five cycles without losing much activity. This indicates a good, sustainable aqueous catalytic application of this catalyst [134].

**Scheme 73:**
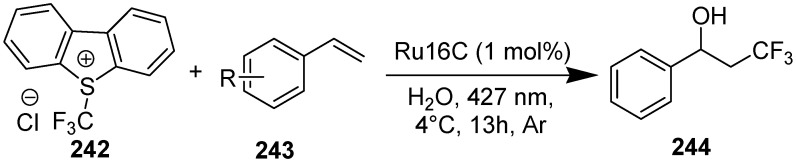
Preparation of *β*-trifluoromethyl alcohols.

In 2025, Wang et al. employed a visible-light-induced photoredox strategy to achieve the hydroxytrifluoromethylation of styrene derivatives (**242**, **243**) in aqueous media. A 1 mol% of a Ru(II)-based photocatalyst (Scheme 73) mediated the reaction between styrene and a CF_3_^+^ reagent under blue LED light in water, and both OH and CF_3_ fragments were added across the double bond of styrene, forming trifluoromethyl alcohols **244** in excellent yields. Several sources of CF_3_^+^ reagents were evaluated, with sulfonium salts providing the best yields with appropriate counter anions [135]. The efficient generation and capture of radicals was facilitated by an aqueous medium, and a controlled radical reaction mediated by the photocatalyst allowed high selectivities under mild, oxygen-free conditions. This provides a greener strategy to perform selective trifluoromethylative alkene functionalization by integrating a photoredox catalytic approach and water.

**Scheme 74:**
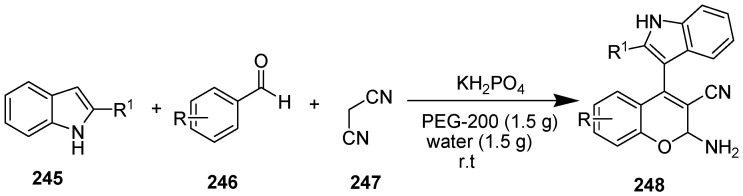
Preparation of 3-substituted indoles.

In 2014, Salih et al. surveyed new progress for the synthesis of 3-substituted indoles **248** using substituted indoles **245**, substituted benzaldehydes **246** and malonitrile **247**, with special attention paid to catalysts, which efficiently promote the key steps with selectivity and an environmentally benign effect. Different roles of bases, Brønsted and Lewis acids, amino acids, ionic liquids, surfactant and heteropoly acid systems in Knoevenagel condensation, Michael addition and Pinner cyclization were reviewed and summarized [56]. Yield and substrate scope were influenced by the catalyst system and reaction media, respectively. Environment-friendly catalysts, e.g., recyclable heterogeneous materials, electrochemistry, and green solvents, were also surveyed (Scheme 74). The article emphasized the scope and utility of these catalysts in the synthesis of pharmacologically important indole compounds and recommended future directions to develop more efficient and greener procedures.

**Scheme 75:**
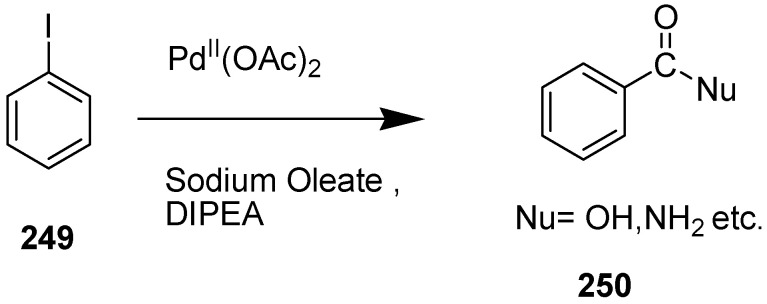
Preparation of substituted benzophenone derivatives.

In 2024, Ravi Pratap Singh et al. reported surfactant-assisted palladium-catalyzed hydroxycarbonylation of aryl iodides in water mediated by the micellar solubilizing role of sodium oleate under a CO atmosphere. Using Pd(OAc)_2_ (1 mol%) and DIPEA (2 eq.) in 5% aq. sodium oleate (0.16 M) at room temperature and 1 atm. CO, aryl iodides readily underwent efficient conversion into corresponding carboxylic acids in a few hours, and for the model substrata, 1-iodo-4-nitrobenzene **249** provided the substituted benzophenone derivatives **250** in excellent isolated yield up to 90% (Scheme 75). A lack of surfactant yielded remarkably slow reactions, while sodium oleate effectively solubilized the hydrophobic substrates and accelerated the reactivity in the gas–liquid–organic triphasic system [46]. The method exhibited good functional group compatibility and could be readily modified to furnish esters and amides. It was found that micellar solubilization and colloidal stabilization of Pd species were responsible for the increased catalytic activity.

**Scheme 76:**
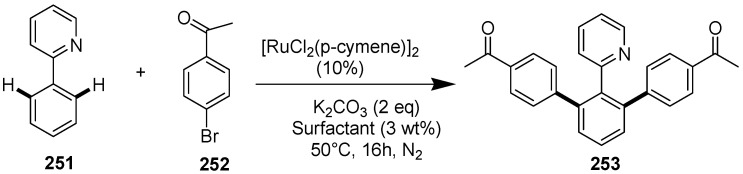
Preparation of a micellar environment enabled efficient ortho-C-H arylation.

In 2024, Hauk et al. developed a next-generation “C-H-friendly” functional surfactant, PyOH-750-M, for Ru-catalyzed direct C-H arylation under micellar conditions in water. By incorporating a pyridone ligand into a PS-750-M-type surfactant, the system overcame compartmentalization issues and promoted efficient metalation, enabling selective C-C bond formation at remarkably mild temperatures (as low as 35 °C). Using substrates such as 2-phenylpyridine **251** as the directing-group-containing arene and aryl halides **252** (e.g., bromo-substituted arenes) as coupling partners in the presence of a Ru(II) catalyst, the tailored micellar environment enabled efficient *ortho*-C-H arylation **253** in water (Scheme 76) [136]. The tailored micellar environment delivered high conversions and excellent chemoselectivity across a broad substrate scope, while conventional surfactants showed much lower activity under similar conditions.

## 4. Future of Surfactants: Surfactants and Surfactant-Based Catalysts as the Future of Organic Chemistry

From a nature perspective, the chemical reactions involved in various synthetic chemistry reactions, including harsh reaction conditions, dangerous substrates and solvents, and heterogeneous catalysts (that require a tedious and long procedure for their preparation), must be carried out by using modern and greener methodologies. Here, we give a comparison of various important reactions that were previously performed by traditional methodologies but are now carried out via clean and green procedures in water.

Comparison between the conventional organic solvent system and surfactant-assisted catalytic methods: A comparison between the conventional organic solvent system and the surfactant-assisted catalytic approach is outlined in Table 1. The reaction was previously carried out using iodine in ethanol with an acceptable result, but the reaction is run in an organic solvent, which does not contribute to sustainability at the conventional level.

In contrast, the reaction is successfully conducted using water Sc(DS)_2_ with much better yield and faster reaction. Performance comparison of conventional methods and the surfactant-assisted catalytic approach: In the Friedlander annulation reaction [**7a**,**b**], a superior efficiency and rate of reaction is observed due to the effect of the formation of micelles, which provide the maximum interaction between substrates and the Lewis acid catalyst by the micellar effect to give better yield. The use of water as a reaction solvent is environmentally favorable.

## 5. Conclusions

Reactions catalyzed by the synthetic organic protocol are expensive, toxic, and also release hazardous materials. To overcome such issues, organic transformations were performed under aqueous conditions, as water-mediated reactions are environmentally benign, non-poisonous, economical, easily accessible, and inexpensive. To make this strategy more efficient and environmentally benign, researchers initiated the use of surfactants and surfactants combined with catalysts, such as LASCs and BASCs, so there is no need to apply hazardous solvents and catalysts. The attractive features of this strategy are straightforward work-ups, natural amiability, as we use water as a solvent, great yields, and shorter reaction times. Further, we have compiled famously named reactions in a tabular form to compare the traditional approaches and current synthetic trends using these surfactants. The broad application of water-mediated reactions, surfactants and LASCs/BASCs catalysts is the driving force for the future scope of this surfactant-applied strategy.

## Figures and Tables

**Table 1:** Comparison of different named reactions with organic solvent systems and surfactant-based systems.

Sr. No.	Name of Reaction	Synthesis via Organic Solvents and a Catalyst	Synthesis via Surfactant-Based Catalyst
**1**	Friedlander annulation	Molecular iodine–ethanol [137]	Sc (DS)_3_/water [38]
**2**	Pechmann reaction	N,N,N-trimethyl-N-propanesul-fonic acid ammonium hydrogen sulfate [TMPSA][HSO_4_/water [138], NaHSO_4_.SiO_2_ and silica chloride/solvent-free conditions [139], FeF_3_/solvent free(MW) [140], Fe_3_O_4_@Boehmite-NH_2_-CoII NPs/solvent-free [141]	NTDSS/water [49]
**3**	Esterification reaction	Ammonium carbonate/water [142]	LASC Ce[LS]_3_/water [51]
**4**	Allylation reaction, alkylation reaction	L-Proline/water [137], InI/THF [143]	Ln(DS)_3_/water [33,60], LASC-BASC/water [43], Calix [5] arene sulfonic acid/water [64]
**5**	Michael reaction	La-NR-linked-BINOL-THF [144]	[STDS]/water [37]
**6**	Aldol reaction	TiCl_4_, CH_2_Cl_2_ [145,146], Yb(OTf)_3_ [147], BaB, DME [148], H_2_O_._THF, (H_2_O-THF-toluene) [25]	LASCs/BASCs catalyst [43], STDS/water [36], [Sc(OTf)_3_ + SDS] [34]
**7**	Bignelli reaction	Yb(OTf)_3_/solvent-free [149]	DBSA/water [62], Ce[LS]_3_/water [50]
**8**	Mannich reaction	*p*-TsOH [150]	DBSA/water [125]
**9**	Pictet–Spengler reaction	Silica gel, EtOH [151], i-PrOH [152]	Perfluorooctane sulfonic acid/water [153]
**10**	Diels–Alder reaction	Moisture-Stable Dialkylimidazolium Salt/DCM [154]	Cu(DS)_2_/water [155]
**11**	Sonogashira coupling	Pd(PPh_3_)_2_Cl_2_, CuI, and triethylamine in THF [156]	Pd(PPh_3_)_2_Cl_2_, CuI, SDS, CTAB/water [157]
**12**	Suzuki–Miyaura cross-coupling	PdCl_2_(dppf)‚CH_2_Cl_2_ catalyst, *i*-PrOH-H_2_O [158]	TPGS-750-M in water [159]
**13**	Heck coupling reaction	Pd catalyst, DMF [160]	CTAB-H_2_O [161]
**14**	Etherification	An acid catalyst, ethanol [162]	DBSA/water [163]
**15**	Pfitzinger reaction	Base KOH, ethanol [164]	CTAOH cetyltrimethylammonium hydroxide [165]
**16**	Hantzsch reaction	Organocatalyst/NH_4_OAc [166], Yb(OTf)_3_/ethanol [167]	Al(DS)_3_, water [168]

## Data Availability

There are no restrictions on materials and data availability.
